# Psoriasis and cardiovascular disease risk in European and East Asian populations: evidence from meta-analysis and Mendelian randomization analysis

**DOI:** 10.1186/s12916-022-02617-5

**Published:** 2022-11-01

**Authors:** Liming Zhang, Yuxiang Wang, Li Qiu, Jian Wu

**Affiliations:** 1 Department of Dermatology, The First Hospital of China Medical University, National Health Commission Key Laboratory of Immunodermatology, Key Laboratory of Immunodermatology of Ministry of Education, No. 155 Nanjing Bei Street, Shenyang, 110001 China; 2 China Mobile Communications Group Co, Ltd, Shenyang, China

**Keywords:** Cardiovascular disease, Psoriasis, Mendelian randomization

## Abstract

**Background:**

Psoriasis has been linked to cardiovascular disease (CVD), including coronary artery disease (CAD), myocardial infarction (MI), and heart failure (HF). However, available studies regarding this relationship have shown inconsistent results. Therefore, in this report, we performed a comprehensive review of the literature to assess the effects of psoriasis on risk of these CVDs.

**Methods:**

A search of literature until 24 December 2021 was done in PubMed, the Cochrane Library, Web of Science, Google Scholar, and Embase. Within European and East Asian populations, meta-analyses of observational studies assessing correlations between psoriasis and various CVD risk factors were conducted. Mendelian randomization (MR) was then employed to assess the causative impact of genetic pre-disposition for psoriasis on these CVD risk factors.

**Results:**

The results of the meta-analyses indicated that, in both the European and East Asian populations, psoriasis was significantly linked to an elevated risk in the incidence of CAD (RR = 1.51, 95% confidence interval (CI): 1.04–2.18, *p* = 0.028 and RR = 1.91, 95% CI: 1.62–2.25, *p* < 0.001) and MI (RR = 1.23, 95% CI: 1.04–1.46, *p* = 0.017 and RR = 2.17, 95% CI: 1.44–3.28, *p* < 0.001). A positive genetic relationship of psoriasis with CAD was found in European individuals (IVW OR:1.03; 95% CI: 1.01–1.06, *p* = 0.005) and in East Asian individuals (IVW OR:1.18; 95% CI: 1.03–1.32, *p* = 0.031). We also established that psoriasis was causally linked with an elevated risk of MI (IVW OR:1.05; 95% CI: 1.01–1.09, *p* = 0.026) in the European population as determined using an MR approach. Moreover, our MR results were congruent with the null findings from the meta-analysis assessing associations of psoriasis with HF risk.

**Conclusions:**

This research work provides preliminary evidence that psoriasis and CVD have a common genetic origin and that targeted psoriasis treatment might improve cardiovascular outcomes. These results not only increase our knowledge of the genetic underpinnings linking a comorbidity of psoriasis with CVD but also suggests a novel approach for CVD prevention.

**Supplementary Information:**

The online version contains supplementary material available at 10.1186/s12916-022-02617-5.

## Background

Psoriasis constitutes a chronic, inflammatory skin disease with an immune-genetic basis that has been linked to numerous diseases, including metabolic syndrome, cancer, as well as cardiovascular disease (CVD). Psoriasis may also be caused by genetic polymorphisms, which vary between Caucasians and Asians [1], as approximately 1–5% of the Western population is affected with psoriasis, whereas this incidence is thought to be less than 1% in the Asian population [2]. Psoriasis is linked with an elevated incidence of cardiovascular (CV) risk, as based on a large body of epidemiological evidence. While there is evidence indicating a higher prevalence of CVD in individuals with psoriasis, this relationship has been fraught with inconsistent findings. In specific, results from previous epidemiological analyses of correlations between psoriasis and CV events (CVEs) have been inconsistent. While a link between psoriasis and major CVEs, for instance myocardial infarction (MI) and coronary artery disease (CAD), along with heart failure (HF) has been reported [3–5], other investigators have reported that psoriasis is not an independent predictor of CVEs and coronary atherosclerosis [6, 7]. Such contradictory findings may be due to variations in research design, psoriasis severity, and confounders along with impact modifiers [8].

Mendelian randomization (MR) is a novel and increasingly popular approach for establishing exposure–outcome links by employing genetic variations as instrumental variables [9]. MR provides a means for enhancing the validity of causal inferences relative to traditional epidemiological research, particularly in the face of confounding factors along with reverse causality [10]. For example, MR has recently been utilized to offer insights into causative links between serum calcium contents, plasma phospholipid arachidonic acid, and tumor necrosis factor and cardiovascular diseases [11–13].

In this current report, we performed meta-analyses of observational studies to describe the relationships of psoriasis with CVE risk in European and East Asian populations in order to determine if the relationship of psoriasis with CVE risk is congruent with causal associations. Subsequently, we conducted a two-sample MR to assess the evidence for a causal association.

## Methods

### Meta-analysis

#### Literature search

Preferred Reporting Items for Meta-Analyses standards [14] were followed in the screening PubMed, Google Scholar, Web of Science, and Embase along with the Cochrane Library by December, 2021. The search strategy (Additional file 1: Table S1) was adapted to the format of each database. The reporting of this study was based on the Preferred Reporting Items for Systematic Review (PRISMA) reporting guidelines (Additional file 2: PRISMA 2020 Checklist). The review was not registered. In addition, a manual reviewing of the reference lists of all relevant articles was conducted to identify any other relevant literature.

#### Inclusion and exclusion criteria

For inclusion, studies are needed to be in English and incorporate the possibility for a connection between psoriasis and risk of CAD, MI, and HF in the general population, along with providing enough data to allow for an assessment of the rate of incidence for CAD, MI, and HF in both psoriasis and control subjects and control groups matching with cardiovascular risk factors of psoriasis patients or adjusted for cardiovascular risk factors.

The following comprised the criteria for exclusion: (I) reviews, guidelines, meta-analyses, editorials, case reports with less than five cases, comments, letters to the editor, and other communications that did not include original data, (II) abstracts from conferences, and (III) animal or in vitro studies.

#### Data extraction and quality assessment

Two reviewers (LMZ and YXW) extracted data from the Microsoft Excel spreadsheet using a standardized data extraction checklist. This form included the primary author, year of publication, study design, sample size of psoriasis cases, sample size of controls, number of CVDs in psoriasis cases, number of CVDs in controls, and associated risk factors of CVD (sex, age, hypertension, dyslipidemia, diabetes, BMI). The third and fourth authors (LQ and JW) were brought in to resolve a disagreement during the extraction process and control groups matching with cardiovascular risk factors of psoriasis patients or adjusted for cardiovascular risk factors.

The Newcastle–Ottawa Scale was adopted to assess the study quality [15]. Each article was assigned a score of up to 9 points on the basis of the quality of population selection (0–4), group comparability (0–2), and exposure evaluation (0–3).

#### Statistical analyses

The meta-analysis was conducted using STATA 16.0. For a more accurate assessment of apparent relationships of psoriasis with CAD, MI, and HF, categorical meta-analyses were used. Relative risk (RR) with a 95% CI was adopted to assess dichotomous outcomes. The Cochran’s *Q* test (*p* < 0.05) and the *I*^2^ statistic were adopted to assess heterogeneity among enrolled articles. The random-effect approach was adopted when *I*^2^ > 50% or when a *p* < 0.05 indicated a high degree of heterogeneity across the articles. A fixed-effect model was applied in the meta-analysis when *I*^2^ was less than 50% or the *p* ≥ 0.05. Subjects in these studies were stratified into sub-groups on the basis of race. A funnel plot, Begg’s test, and Egger’s test were employed to assess the likelihood of publication bias. Sensitivity analyses were performed by successively excluding each article in order to determine the strength of the primary conclusions.

### Mendelian randomization

#### Study design and data sources

**Fig. 1:**
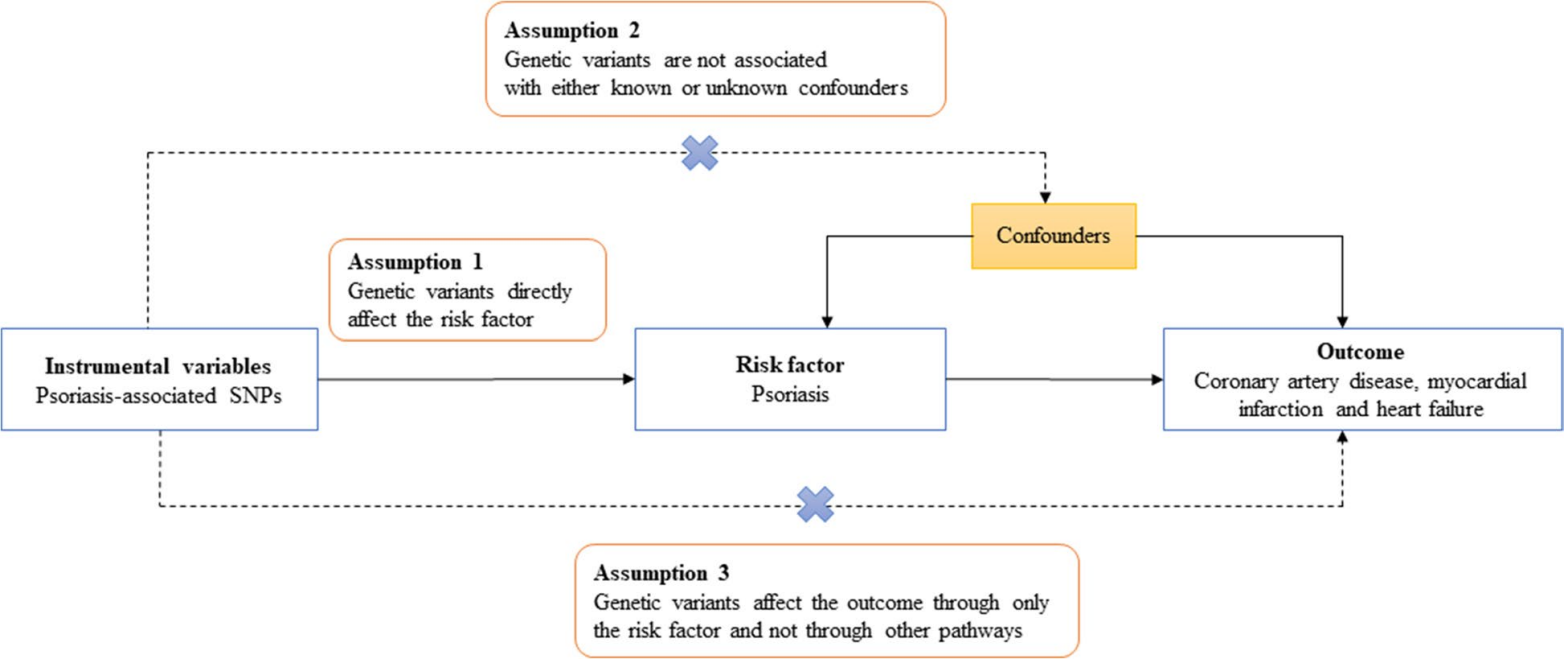
Schematic diagram illustrating Mendelian randomization assumptions underlining a Mendelian randomization analysis on the relationship of psoriasis with coronary artery disease, myocardial infarction, and heart failure

The Mendelian randomization (MR) method is based on three main assumptions as summarized in Fig. 1. First, the risk factor should be associated with the genetic variations used as instrumental variables. Second, confounders should not be associated with genetic variations. Third, genetic polymorphisms should influence outcome risk via the risk factor and not through additional routes [11].

Initially, SNPs associated with European [16–25] or East Asian [26–29] psoriasis at the genome-wide significance threshold (*p* < 5 × 10^−8^) by the NHGRI-EBI GWAS catalog, MRBASE platform, and published studies were extracted. These SNPs were then clumped via the PLINK clumping algorithm to ensure that each selected SNP was independent (*r*^2^ < 0.001). Linkage disequilibrium correlations between the European and East Asian psoriasis SNPs were assessed on the basis of the European or East Asian ancestry reference panel (1000 Genome Project), respectively. Each psoriasis-linked SNP was investigated for pleiotropic relationships with possible confounders, including lipids, glycemic traits, type 2 diabetes, body mass index (BMI), adjusted waist-to-hip ratios, and systolic/diastolic blood pressures.

Summary statistics for European CAD and MI were abstracted from the Coronary Artery Disease Genome-Wide Replication and Meta-analysis plus the Coronary Artery Disease Genetics (CARDIoGRAMplusC4D) consortium. Summary statistics for CAD were taken from a study that included a meta-analysis of genetic associations for CAD from the UK Biobank and CARDIoGRAMplusC4D involving 122,733 cases and 424,528 controls of European ancestry [30]. Summary statistics for MI were taken from a study that included a meta-analysis of the Exome-chip research works of European descent focusing on 42,335 patients and 78,240 controls [31, 32]. Summary statistics for European HF were extracted from the Heart Failure Molecular Epidemiology for Therapeutic Targets (HERMES) consortium based on a genome-wide association of 26 meta-analysis studies involving 47,309 cases along with 930,014 controls of European ancestry [33].

Summary statistics for East Asian CAD, MI, and HF were extracted from the NHGRI-EBI GWAS catalog. Summary statistics of CAD, MI, and HF consisted of 15,302 CAD patients and 36,140 controls [34], 14,992 MI patients and 146,214 controls [35], and 10,540 HF patients and 168,186 controls [35].

#### Statistical analysis

To quantify the influence of genetically predicted psoriasis on CAD, MI, and HF susceptibility, a two-sample MR was utilized. The TwoSampleMR R package (https://mrcieu.github.io/TwoSampleMR/) was adopted to accomplish MR along with sensitivity analyses, with effect estimates compared across five distinct methods: inverse-variance weighted (IVW), MR-Egger, weighted median, weighted mode, and simple mode. As each of these approaches makes distinct assumptions about the nature of pleiotropy, agreement in the point estimate across methods was used to enhance causal evidence. The MR Steiger test was also performed to infer the causal direction between psoriasis and CAD, MI and HF. With this test, it is possible to calculate the variance explained in the exposure and outcomes by the instrumenting SNPs, and it also tests if variance in the outcome is less than the exposures. The Cochran *Q* statistic was adopted to assess the variability in IVW estimations between SNPs. To assess the influence of particular SNPs on the summary estimates, a ‘leave-one-out’ approach was adopted. An inclusion of the intercept in the regression analysis was adopted to assess horizontal pleiotropy via a Mendelian randomization Egger. We adopted a 0.017 ([*p* < 0.05]/3 outcome measures) as a conservative Bonferroni-based *p* threshold value. Associations with a *p* value between the Bonferroni-corrected significance level and the conventional significance level (*p* < 0.05) signified suggestive relationships.

## Results

### Observed associations between psoriasis and CAD, MI, and HF risk.

**Fig. 2:**
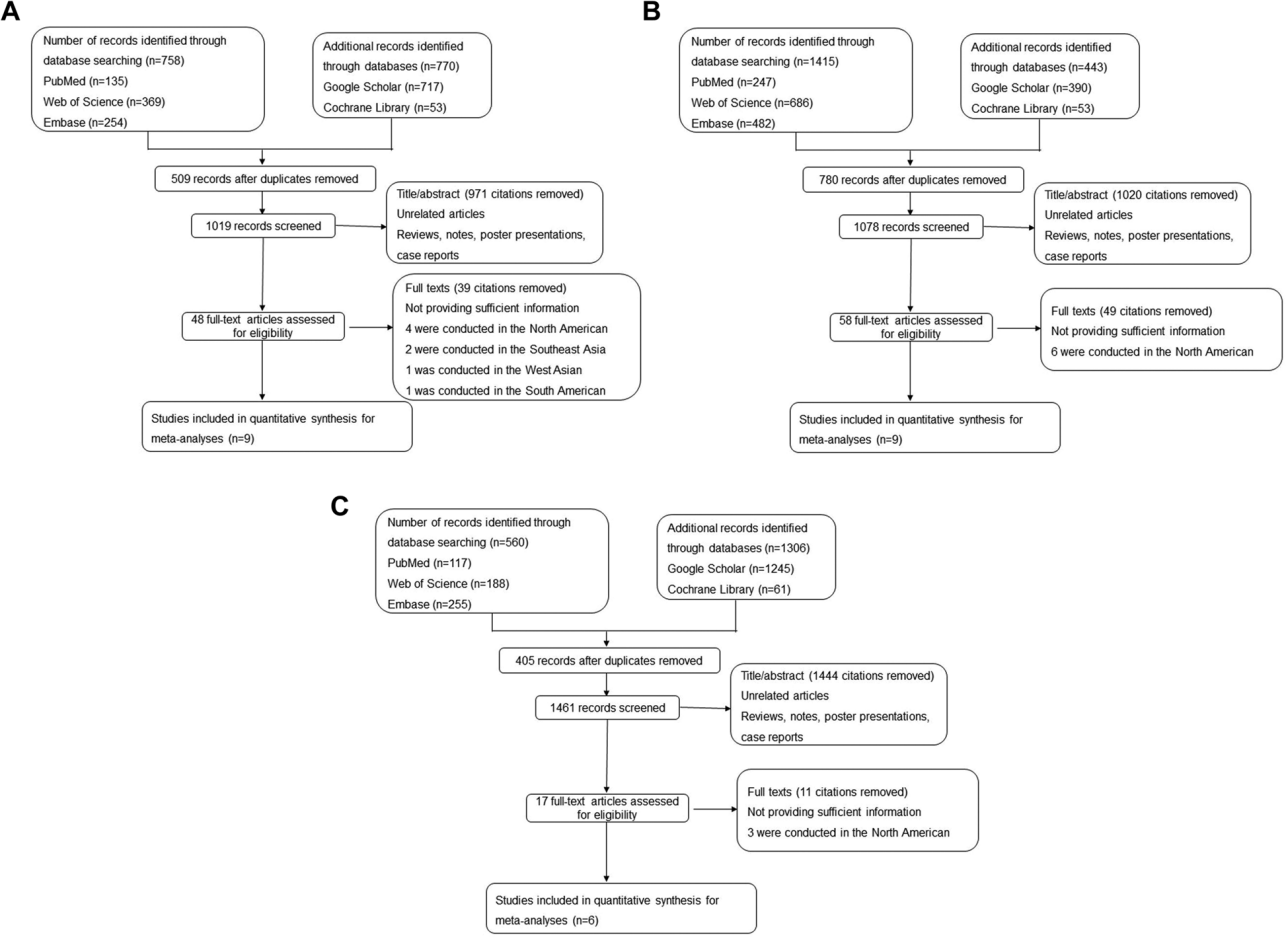
Flow chart illustrating details regarding the literature selection for CAD (**a**), MI (**b**), and HF (**c**). CAD, coronary artery disease; MI, myocardial infarction; HF, heart failure

**Fig. 3:**
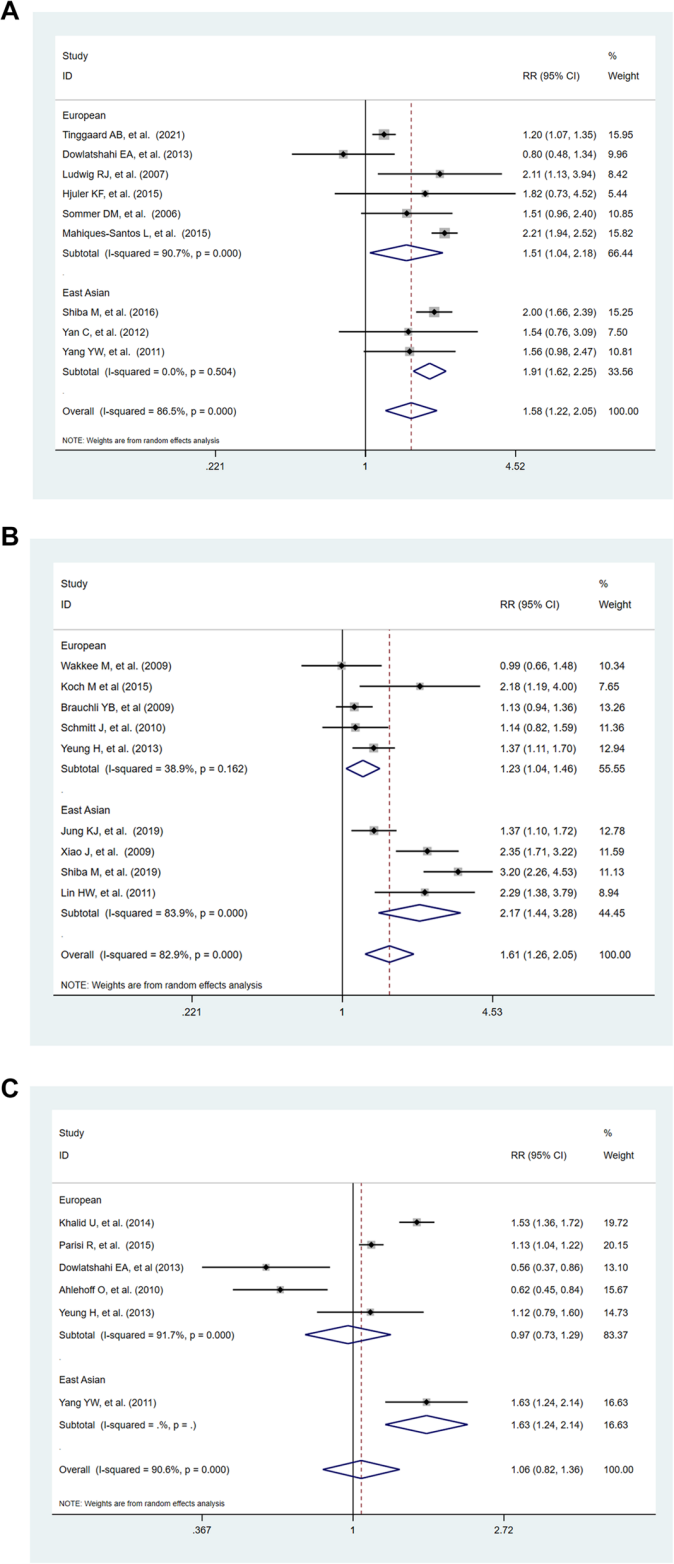
Meta-analyses on the relationship of psoriasis with CAD (**a**), association between psoriasis and MI (**b**), and HF (**c**). CAD, coronary artery disease; MI, myocardial infarction; HF, heart failure

Figure 2 is a flow chart exhibiting the process of the detailed literature selection. Direct transcripts of the executed search strategies were listed in appendix data (Additional file 3, Additional file 4 and Additional file 5). A total of 9 CAD articles [3, 6, 36–42], 9 MI articles [2, 4, 43–49], and 6 HF articles [5, 6, 42, 48, 50, 51] were ultimately enrolled in the three meta-analyses after screening, respectively. With regard to studies from Europe versus East Asia, there were, respectively, 6 versus 3 CAD studies, 5 versus 4 MI studies, and 5 versus 1 HF studies. Characteristics and methodological quality of the 3 meta-analyses for these studies are provided in Additional file 1: Table S2. Within the European population, psoriasis was remarkably associated with a higher risk incidence of CAD (RR = 1.51, 95% CI: 1.04–2.18, *p* = 0.028, Fig. 3a) and MI (RR = 1.23, 95% CI: 1.04–1.46, *p* = 0.017, Fig. 3b), while the risk incidence of HF (RR = 0.97, 95% CI: 0.93–1.29, *p* = 0.816, Fig. 3c) was not significantly increased with psoriasis. In the East Asian population, psoriasis was significantly associated with a higher risk of CAD (RR = 1.91, 95% CI: 1.62–2.25, *p* < 0.001, Fig. 3a) and MI (RR = 2.17, 95% CI: 1.44–3.28, *p* < 0.001, Fig. 3b). No heterogeneity among studies was detected in all analyses. Sensitivity analysis of the meta-analyses regarding psoriasis and CAD, MI, and HF risk revealed that the pooled data were not driven by a single study (Additional file 1: Figure S1).

### Genetic associations between psoriasis and CAD, MI, and HF risk

A total of 97 GWAS-significant SNPs (*p* < 5 × 10^−8^, Additional file 1: Table S3) associated with psoriasis in European ancestry were reported by the NHGRI-EBI GWAS catalog, MRBASE platform, and published studies. Of these 97 SNPs associated with psoriasis, 54 variants demonstrated pleiotropic relationships at the Bonferroni-corrected significance threshold (*p* < 0.05/98 SNPs = 5.1 × 10^−4^) with type 2 diabetes, BMI, CAD, HF, and diastolic/systolic blood pressures (Additional file 1: Table S4). Pleiotropic SNPs were deleted to avoid violating the Mendelian randomization assumptions (Fig. 1). Of the 43 SNPs, 25 were significantly correlated (*r*^2^ ≥ 0.001). Thus, 18 SNPs associated with psoriasis in European ancestry were employed as instrumental variables in the MR analyses (Table 1). We found that genetically predicted psoriasis was causally linked with an increased risk of CAD (IVW OR:1.03; 95% CI: 1.01–1.06, *p* = 0.005), suggestive of a relationship with an elevated risk of MI (IVW OR:1.05; 95% CI: 1.01–1.09, *p* = 0.026), but no convincing support for any association with HF risk (IVW OR:1.03; 95% CI: 0.99–1.07, *p* = 0.106) was obtained in this European population using IVW (Table 2 and Additional file 1: Figure S2). Results of the *Q*-statistic suggested no heterogeneity (IVW: *Q* = 4.66, *p* = 0.990, MR-Egger: *Q* = 4.64, *p* = 0.982), and the MR-Egger intercept (intercept = 0.0006, *p* = 0.896) suggested no pleiotropy for psoriasis and CAD risk, there was no any significant heterogeneity (IVW: *Q* = 11.19, *p* = 0.798, MR-Egger: *Q* = 11.03, *p* = 0.750) or pleiotropy (intercept = 0.003, *p* = 0.699) present for psoriasis and MI risk (Additional file 1: Table S5).

**Table 1:** Psoriasis associated SNPs were used as instrumental variables in the Mendelian randomization analyses

Instrumental variables with psoriasis of European ancestry				Instrumental variables with psoriasis of East Asian ancestry			
SNP	Beta	SE	*P*	SNP	Beta	SE	*P*
rs240993	0.223	0.024	5.00E − 20	rs5063	− 0.163	0.028	3.51E − 09
rs702873	0.113	0.019	4.00E − 09	rs41268474	0.157	0.024	6.00E − 11
rs28512356	0.157	0.029	4.00E − 08	rs2276405	− 0.186	0.031	3.00E − 09
rs9533962	0.113	0.017	8.00E − 11	rs72933970	0.148	0.026	1.00E − 08
rs10789285	0.113	0.019	3.00E − 09	rs2781377	− 0.163	0.025	4.00E − 11
rs4685408	0.113	0.015	7.00E − 14	rs11544355	− 1.772	0.272	7.00E − 11
rs7637230	0.131	0.021	2.00E − 10	rs9808753	− 0.083	0.015	3.00E − 08
rs11053802	0.104	0.018	4.00E − 09	rs6444895	0.104	0.015	1.00E − 12
rs118086960	0.113	0.02	7.00E − 09	rs149442660	− 3.219	0.468	6.00E − 12
rs12118303	0.113	0.018	3.00E − 10	rs143700362	− 3.507	0.391	3.00E − 19
rs41298997	0.122	0.022	2.00E − 08	rs2233278	0.673	0.108	4.00E − 10
rs76959677	0.247	0.045	3.00E − 08	rs249038	− 0.174	0.031	2.00E − 08
rs9513593	0.113	0.021	4.00E − 08	rs149798287	− 0.994	0.173	1.00E − 08
rs2700987	0.104	0.018	4.00E − 09	rs144706178	− 2.207	0.278	2.00E − 15
rs9504361	0.113	0.017	2.00E − 11	rs4141001	− 0.151	0.022	2.00E − 11
rs28998802	0.199	0.024	3.00E − 16	rs2778031	− 0.186	0.015	1.00E − 36
rs9988642	0.419	0.039	1.00E − 26	rs1050414	1.161	0.155	6.00E − 14
rs10865331	0.113	0.018	5.00E − 10	rs12884468	− 0.128	0.022	1.00E − 08

*SNP* single nucleotide polymorphism, *SE* standard error

**Table 2:** Results of the MR analyses inferring causality of psoriasis upon CAD, MI and HF risk

Trait	MR Method	MR Result in Europeans		MR Result in East Asian	
		Beta (SE)	*P*	Beta (SE)	*P*
CAD	Inverse variance weighted	0.033 (0.012)	**0.005**	0.161 (0.075)	**0.031**
	MR Egger	0.029 (0.030)	0.359	− 0.074 (0.126)	0.569
	Weighted median	0.022 (0.015)	0.141	0.028 (0.098)	0.772
	Simple mode	0.017 (0.025)	0.498	0.177 (0.238)	0.472
	Weighted mode	0.019 (0.020)	0.356	0.047 (0.087)	0.602
MI	Inverse variance weighted	0.047 (0.021)	**0.026**	− 0.036 (0.022)	0.099
	MR Egger	0.025 (0.059)	0.674	− 0.064 (0.032)	0.069
	Weighted median	0.058 (0.027)	0.035	− 0.019 (0.032)	0.550
	Simple mode	0.066 (0.048)	0.190	− 0.048 (0.054)	0.385
	Weighted mode	0.061 (0.036)	0.113	− 0.043 (0.028)	0.144
HF	Inverse variance weighted	0.031 (0.019)	0.106	− 0.032 (0.024)	0.181
	MR Egger	0.014 (0.051)	0.789	− 0.042 (0.035)	0.255
	Weighted median	0.023 (0.024)	0.325	− 0.035 (0.031)	0.265
	Simple mode	− 0.002 (0.036)	0.960	− 0.042 (0.056)	0.467
	Weighted mode	0.022 (0.031)	0.476	− 0.039 (0.030)	0.227

*MR* Mendelian randomization, *CAD* coronary artery disease, *MI* myocardial infarction, *HF* heart failure, *SE* standard error

For the East Asian population, 33 GWAS-significant SNPs (*p* < 5 × 10^−8^, Additional file 1: Table S6) associated to psoriasis were reported within the NHGRI-EBI GWAS catalog, MRBASE platform, and published studies. Of these 33 SNPs associated with psoriasis, 8 variants demonstrated pleiotropic relationships at the Bonferroni-corrected significance threshold (*p* < 0.05/33 SNPs = 0.0015) with type 2 diabetes, BMI, ischemic stroke, and CAD (Additional file 1: Table S7). Of the 25 SNPs, 7 SNPs were correlated (*r*^2^ ≥ 0.001). Thus, 18 SNPs associated with psoriasis in East Asian ancestry were adopted as instrumental variables in the MR analyses (Table 1). There were suggestive associations of genetically predicted psoriasis with an elevated risk of CAD (IVW OR: 1.18; 95% CI: 1.03–1.32, *p* = 0.031). There was no genetically predicted psoriasis associated with MI (IVW OR: 0.96; 95% CI: 0.92–1.00, *p* = 0.099) or HF (IVW OR: 0.97; 95% CI: 0.92–1.02, *p* = 0.181) risk in this East Asian population (Table 2 and Additional file 1: Figure S2). Results of the *Q*-statistic suggested no heterogeneity (IVW: *Q* = 10.02, *p* = 0.528, MR-Egger: *Q* = 4.65, *p* = 0.913), and the MR-Egger intercept (intercept = 0.054, *p* = 0.049) suggested no pleiotropy for psoriasis and CAD risk (Additional file 1: Table S5).

The inferred causal direction between exposure (psoriasis) and outcomes (CAD, MI, and HF) were “TRUE” as based on our MR Steiger test (Additional file 1: Table S8).

From the “leave-one-out” assessment, no single SNP was identified as remarkably influencing the results (Additional file 1: Figure S3).

## Discussion

To our knowledge, this is the first report employing meta-analysis and MR to investigate causative associations between psoriasis and CAD, MI, and HF risk in European and East Asian populations. In our observational analyses, we found that psoriasis was remarkably associated with a higher risk of incident CAD and MI and was not associated with HF risk. In order to eliminate the potential for interference of cardiovascular risk factors in the analysis of correlations between psoriasis and CAD, MI, and HF, only correlations between psoriasis and CAD, MI and HF were included in the analysis, while correlations between psoriasis and peripheral vascular disease, atherosclerosis, and other cardiovascular diseases or cardiovascular risk factors were not analyzed. Moreover, in the studies included in this report, control groups were closely matched with cardiovascular risk factors of psoriasis patients or adjusted for cardiovascular risk factors. With MR, we systematically evaluated the causality between psoriasis and CAD, MI, and HF risk and found that psoriasis was linked with a higher risk of CAD in both European and East Asian populations. Besides, we observed that psoriasis was causally linked to an elevated risk of MI in European population using an MR approach. There was a difference between the relative risk as obtained with observational analyses versus that with MR. Although attempting to exclude the influence of other coexisting risk factors in observational studies, confounding factors could not be completely eliminated, as the meta-analysis studies were not randomized controlled studies, which are widely accepted as the gold standard for addressing the issue of causality. All genetic variants associated with the confounders of CAD, MI, and HF were excluded in our study. While we used the most comprehensive set of genetic variants currently available, these variants accounted for only a portion of the psoriasis variance across individuals. Therefore, it is possible that some unknown psoriasis-related SNPs may also play an important role in the development of AD, MI, and HF. In fact, the reason for the lower relative risk as obtained with the MR versus observational study might, in part, be attributable to these factors as described above.

The effects of psoriasis on CAD were consistent within both the European and East Asian populations, when causative patterns of the three risk variables were evaluated across the two ancestries. When assessing the influence of psoriasis with MI, however, distinct causation patterns across ancestries were identified, with a suggestive causal estimate in Europeans that was not reflected in the analyses of East Asians. These results suggest that interventions for MI risk factors in one ancestry require careful consideration prior to implementation within another ancestry.

**Fig. 4:**
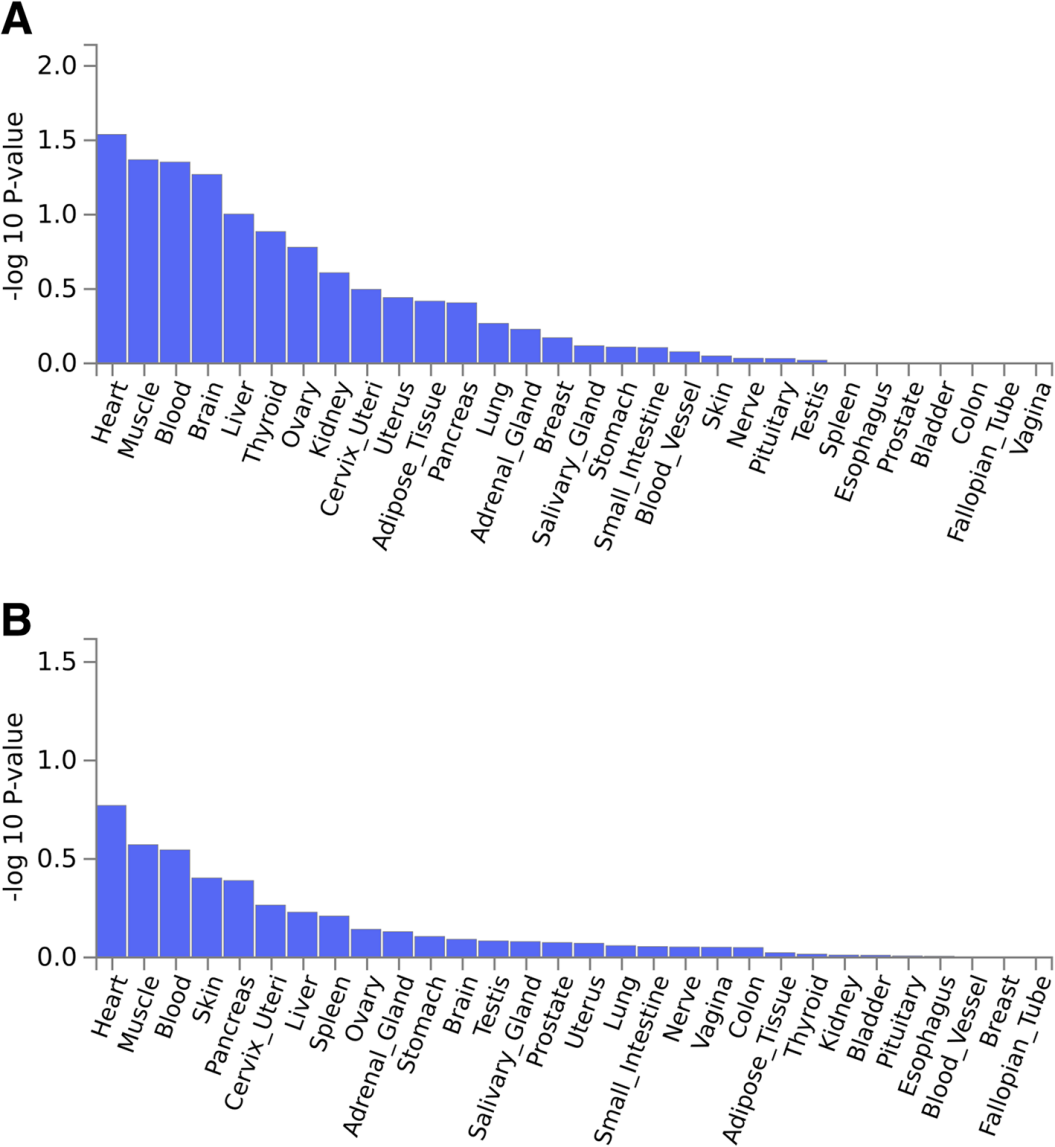
GTEx tissue enrichment analysis for expression of psoriasis loci in European (**a**) and East Asian (**b**) populations

Expression quantitative trait loci (eQTL) analyses were performed to examine the relationship between European versus East Asian instrumental SNPs for MR and gene expression using SNP2GENE function of Functional Mapping and Annotation of Genome-wide Association Studies (FUMA GWAS). Enrichment analysis for gene sets as obtained from the differential eQTL analyses was performed using GENE2FUNC function of FUMA GWAS and the tissue-specific gene expression patterns as based on GTEx v8 54. Most psoriasis loci were found to be remarkably expressed in the heart, brain, and blood (Fig. 4), indicating that these features might involve a malfunction of the cardiovascular system. Some psoriasis loci showed a substantial single-tissue eQTL, primarily in the cardiovascular system (artery coronary: rs240993/*KIAA1919*, rs9808753/*TMEM50B*, heart left ventricle: rs11053802/*KLRC4*, heart left ventricle: rs11053802/*KLRK1*, rs5063/*CLCN6*, rs1050414/*HLA-B*, rs2778031/*SPIN1*). Notably, *KLRC4* belongs to the killer cell lectin-like receptor subfamily C, which has been reported to be enriched in defensive response-linked biological processes and essential for membrane-related cellar components. The primary mechanism of MI development involves immune responses, which are triggered by antigen processing and presentation processes. It has been suggested that KLRC4 is differentially expressed in MI patients [52]. Results from an animal study have demonstrated that blockade of the NK cell lectin-like receptor K1 gene (*KLRK1*, encoding *NKG2D*) combined with CTLA-4–Ig attenuated cardiac allograft vasculopathy, and this influence was linked to a lower alloantibody generation, a repressed IL-6 expression, and a strengthened expansion of modulatory T cells [53]. Many rare coding mutations and intronic single nucleotide polymorphisms in *CLCN6* have been related with decreased blood pressure and hypertension, along with stroke risk as based on recent GWAS findings in humans [54]. *CLCN6* encodes the voltage-gated chloride channel 6 (ClC-6) and influences vascular smooth muscle contractility along with arterial stiffness through changes in Golgi calcium stores [55]. HLA-B*35-linked haplotypes are more prevalent in CAD subjects as compared with that of healthy age- and sex-matched controls [56]. Additionally, miR-489 can enhance cardiac muscle cell apoptosis following ischemia–reperfusion via dampening of the SPIN1-mediated PI3K/AKT cascade [57]. Based on a large GWAS consortia, our MR results present convincing evidence of a causative role for psoriasis in CAD and MI, despite the unknown confounders in observational studies, underlining the importance of treating psoriasis in reducing the risk of the onset of CAD and MI.

This study had some limitations. Firstly, a notable limitation of our study was that it was not stratified by the severity of psoriasis. As the original data were obtained from observational studies, it was not possible to evaluate or clarify the effects of psoriasis severity stratification. Related to this issue, none of the psoriasis-associated SNPs identified were described in association with the severity of psoriasis. Therefore, we were unable to assess the causal impact of CVD risk as related to the stratification of psoriasis severity. Secondly, preprints were not included in our search, as we only searched for peer-reviewed English language publications. English-language restricted meta-analyses and other language-inclusive meta-analyses are probably not significantly different in terms of summary prevalence [58], and results of systematic reviews were not affected by the exclusion of non-English language publications [59]. Thirdly, we excluded some studies that control groups were not matched with cardiovascular risk factors of psoriasis patients or not adjusted for cardiovascular risk factors. This operation might exclude some useful studies, but it also excluded the possibility that psoriasis combined with cardiovascular disease is caused by other confounding factors.

## Conclusions

In summary, from our study, we were able to establish a causal association of psoriasis with CAD and MI. Such findings have important implications as they provide further support for the targeted treatment of psoriasis as a means of enhancing beneficial effects upon cardiovascular outcomes. The possible mechanisms accounting for this causal relationship of psoriasis with CAD and MI require further investigation.

## Supplementary Information

**Additional file 1:** **Table S1.** Search strategy of psoriasis and coronary artery disease. **Table S2.** Characteristics and methodological quality of the qualified studies of the 3 meta-analyses. **Table S3.** SNPs (*p* < 5 × 10^−8^) associated with psoriasis of European ancestry were previous reported. **Table S4.** The pleiotropic psoriasis-associated SNPs with cardiometabolic traits in European ancestry. **Table S5.** Heterogeneity and pleiotropy analysis of the psoriasis on CAD, MI and HF risk. **Table S6.** SNPs (*p* < 5 × 10^−8^) associated with psoriasis of East Asian ancestry were previous reported. **Table S7.** The pleiotropic psoriasis-associated SNPs with cardiometabolic traits in East Asian ancestry. **Table S8.** MR Steiger directionality test. **Figure S1.** Sensitivity analysis of the meta-analyses about psoriasis and CAD risk (a), MI risk (b) and HF risk (c). **Figure S2.** A plot relating the effect sizes of the SNP-psoriasis association (x-axis, log OR) and the SNP-CVD associations (y-axis, log OR) with standard error bars. **Figure S3.** Leave-one-out MR analysis for SNPs used as instruments MR analysis.

**Additional file 2.** The preferred reporting items for systematic review and meta-analysis (PRISMA) 2020 statement.

**Additional file 3.** The direct transcripts of the executed search strategies about CAD and psoriasis.

**Additional file 4.** The direct transcripts of the executed search strategies about MI and psoriasis.

**Additional file 5.** The direct transcripts of the executed search strategies about HF and psoriasis.

## Data Availability

All the data used in the present study had been publicly available, and the source of the data had been described in the main text.

## Abbreviations

CVD: Cardiovascular disease
CAD: Coronary artery disease
MI: Myocardial infarction
HF: Heart failure
MR: Mendelian randomization
RR: Relative risk
BMI: Body mass index
IVW: Inverse-variance weighted
