# Supplementary material for: Psoriasis and cardiovascular disease risk in European and East Asian populations: evidence from meta-analysis and Mendelian randomization analysis
Source: BMC Med. 2022 Nov 1;20:421. doi: 10.1186/s12916-022-02617-5 (PMC9628092; doi:10.1186/s12916-022-02617-5)

**Supplemental material**

**Psoriasis and cardiovascular disease risk in European and East Asian populations: evidence from meta-analysis and Mendelian randomization analysis**

**Supplementary Table 1.** Search strategy of psoriasis and coronary artery disease

| **Database** | **Search Terms** |
| --- | --- |
| PubMed | ((psoriasis[Title/Abstract]) AND (coronary artery disease*[Title/Abstract] OR ischaemic heart disease*[Title/Abstract]) AND (("1966/1/1"[Date - Publication] : "2021/12/31"[Date - Publication])) |
| Web of Science | (TS=(psoriasis)) AND TS=(coronary artery disease* or ischaemic heart disease*) by 1960-2021 |
| Embase | #1 psoriasis:ab,ti AND 'coronary artery disease':ab,ti AND [<1966-2021]/py  #2 psoriasis:ab,ti AND 'ischaemic heart disease':ab,ti AND [<1966-2021]/py  #3 #1 OR #2 |
| Google Scholar | "psoriasis" AND "coronary artery disease" or "ischaemic heart disease" |
| Cochrane Library | #1 (psoriasis):ti,ab,kw AND (coronary artery disease):ti,ab,kw with Publication Year to 2021, in Trials (Word variations have been searched)  #2 (psoriasis):ti,ab,kw AND (ischaemic heart disease):ti,ab,kw with Publication Year to 2021, in Trials (Word variations have been searched)  #3 #1 OR #2 |

**Supplementary Table 2.** Characteristics and methodological quality of the qualified studies of the 3 meta-analyses

| Study | Country | Prevalence of CAD (n/N) | |  | Prevalence of MI (n/N) | |  | Prevalence of HF (n/N) | |  | NOS  score |
| --- | --- | --- | --- | --- | --- | --- | --- | --- | --- | --- | --- |
|  |  | Psoriasis cases | Controls |  | Psoriasis cases | Controls |  | Psoriasis cases | Controls |  |  |
| Sommer DM, et al (2006)^1^ | Germany | 32/581 | 38/1044 |  |  |  |  |  |  |  | 7 |
| Ludwig RJ, et al (2007)^2^ | Germany | 19/32 | 9/32 |  |  |  |  |  |  |  | 7 |
| Dowlatshahi EA, et al (2013)^3^ | Netherlands | 14/259 | 536/7931 |  |  |  |  | 20/259 | 1091/7931 |  | 6 |
| Hjuler KF, et al (2015)^4^ | Denmark | 16/58 | 5/33 |  |  |  |  |  |  |  | 7 |
| Mahiques-Santos L, et al (2015)^5^ | Spain | 417/9181 | 450/21925 |  |  |  |  |  |  |  | 6 |
| Shiba M, et al (2016)^6^ | Japan | 108/1197 | 5059/111868 |  |  |  |  |  |  |  | 6 |
| Yan C, et al (2019)^7^ | China | 20/322 | 12/297 |  |  |  |  |  |  |  | 7 |
| Tinggaard AB, et al (2021)^8^ | Denmark | 232/1192 | 6238/38598 |  |  |  |  |  |  |  | 7 |
| Yang YW, et al (2011)^9^ | China | 27/1685 | 52/5055 |  |  |  |  |  |  |  |  |
| Wakkee M, et al (2009)^10^ | Dutch |  |  |  | 37/15820 | 65/27577 |  |  |  |  | 7 |
| Xiao J, et al (2009)^11^ | China |  |  |  | 215/3092 | 45/1521 |  |  |  |  | 7 |
| Brauchli YB, et al (2009)^12^ | UK |  |  |  | 238/36702 | 211/36702 |  |  |  |  | 7 |
| Schmitt J, et al (2010)^13^ | Germany |  |  |  | 73/3147 | 64/3147 |  |  |  |  | 7 |
| Lin HW, et al (2011)^14^ | China |  |  |  | 22/4752 | 48/23760 |  |  |  |  | 7 |
| Yeung H, et al (2013)^15^ | UK |  |  |  | 95/9035 | 693/90350 |  | 34/9035 | 303/90350 |  | 7 |
| Koch M, et al (2015)^16^ | Germany |  |  |  | 11/199 | 101/3986 |  |  |  |  | 7 |
| Jung KJ, et al (2019)^17^ | Korea |  |  |  | 75/99875 | 16369/29962276 |  |  |  |  | 6 |
| Shiba M, et al (2019)^18^ | Japan |  |  |  | 32/1197 | 934/111868 |  |  |  |  | 6 |
| Ahlehoff O, et al (2010)^19^ | Denmark |  |  |  |  |  |  | 39/36992 | 6842/4003265 |  | 6 |
| Yang YW, et al (2011)^20^ | China |  |  |  |  |  |  | 76/1685 | 140/5055 |  | 7 |
| Khalid U, et al (2014)^21^ | Denmark |  |  |  |  |  |  | 286/66389 | 15169/5376842 |  | 6 |
| Parisi R, et al (2015)^22^ | UK |  |  |  |  |  |  | 719/48523 | 2733/208187 |  | 6 |

*CAD* coronary artery disease, *MI* myocardial infarction, *HF* heart failure, *NOS* Newcastle-Ottawa Scale, *UK* United Kingdom. n: the number of CAD/MI/HF patients in psoriasis patients or controls. N: the number of all psoriasis patients or all controls investigated.

**Supplemental Table 3** SNPs (*p* < 5 X 10^-8^) associated with psoriasis of European ancestry were previous reported

| SNP | Beta | SE | *P* |
| --- | --- | --- | --- |
| rs10789285^ref23^ | 0.113 | 0.019 | 3.00E-09 |
| rs10865331^ref25^ | 0.113 | 0.018 | 5.00E-10 |
| rs11053802^ref24^ | 0.104 | 0.018 | 4.00E-09 |
| rs118086960^ref24^ | 0.113 | 0.02 | 7.00E-09 |
| rs12118303^ref24^ | 0.113 | 0.018 | 3.00E-10 |
| rs240993^ref18^ | 0.223 | 0.024 | 5.00E-20 |
| rs2700987^ref25^ | 0.104 | 0.018 | 4.00E-09 |
| rs28512356^ref22^ | 0.157 | 0.029 | 4.00E-08 |
| rs28998802^ref25^ | 0.199 | 0.024 | 3.00E-16 |
| rs41298997^ref24^ | 0.122 | 0.022 | 2.00E-08 |
| rs4685408^ref23^ | 0.113 | 0.015 | 7.00E-14 |
| rs702873^ref18^ | 0.113 | 0.019 | 4.00E-09 |
| rs7637230^ref23^ | 0.131 | 0.021 | 2.00E-10 |
| rs76959677^ref24^ | 0.247 | 0.045 | 3.00E-08 |
| rs9504361^ref25^ | 0.113 | 0.017 | 2.00E-11 |
| rs9513593^ref24^ | 0.113 | 0.021 | 4.00E-08 |
| rs9533962^ref22^ | 0.113 | 0.017 | 8.00E-11 |
| rs9988642^ref25^ | 0.419 | 0.039 | 1.00E-26 |
| rs2395029^ref16^ | 1.411 | 0.133 | 2.00E-26 |
| rs2201841^ref17^ | 0.122 | 0.022 | 3.00E-08 |
| rs2066808^ref17^ | 0.293 | 0.048 | 1.00E-09 |
| rs4112788^ref18^ | 0.255 | 0.04 | 3.00E-10 |
| rs10484554^ref18^ | 1.539 | 0.049 | 4.00E-214 |
| rs2546890^ref20^ | 0.432 | 0.046 | 1.00E-20 |
| rs1047781^ref21^ | 0.168 | 0.031 | 4.00E-08 |
| rs34394770^ref22^ | 0.148 | 0.027 | 3.00E-08 |
| rs8128234^ref22^ | 0.157 | 0.029 | 4.00E-08 |
| rs114934997^ref23^ | 0.157 | 0.027 | 6.00E-09 |
| rs2675662^ref23^ | 0.113 | 0.015 | 2.00E-14 |
| rs11059675^ref24^ | 0.095 | 0.017 | 2.00E-08 |
| rs11065979^ref24^ | 0.077 | 0.014 | 2.00E-08 |
| rs28624578^ref24^ | 0.166 | 0.027 | 9.00E-10 |
| rs2944542^ref24^ | 0.077 | 0.014 | 2.00E-08 |
| rs34517439^ref24^ | 0.166 | 0.028 | 4.00E-09 |
| rs492602^ref24^ | 0.104 | 0.015 | 7.00E-13 |
| rs559406^ref24^ | 0.095 | 0.015 | 1.00E-10 |
| rs61871342^ref24^ | 0.095 | 0.016 | 2.00E-09 |
| rs10979182^ref25^ | 0.113 | 0.02 | 2.00E-08 |
| rs3802826^ref25^ | 0.113 | 0.019 | 1.00E-09 |
| rs545979^ref25^ | 0.113 | 0.018 | 4.00E-10 |
| rs963986^ref25^ | 0.14 | 0.024 | 5.00E-09 |

Supplemental Table 3 continued

| SNP | Beta | SE | *P* |
| --- | --- | --- | --- |
| rs11795343^ref25^ | 0.104 | 0.016 | 8.00E-11 |
| rs12720356^ref25^ | 0.223 | 0.035 | 3.00E-10 |
| rs4561177^ref25^ | 0.131 | 0.018 | 8.00E-13 |
| rs8016947^ref25^ | 0.148 | 0.018 | 3.00E-17 |
| rs1056198^ref25^ | 0.148 | 0.019 | 2.00E-14 |
| rs12445568^ref25^ | 0.148 | 0.018 | 1.00E-16 |
| rs1295685^ref25^ | 0.166 | 0.026 | 3.00E-10 |
| rs2066819^ref25^ | 0.329 | 0.039 | 5.00E-17 |
| rs2111485^ref25^ | 0.131 | 0.024 | 3.00E-08 |
| rs4821124^ref25^ | 0.122 | 0.022 | 4.00E-08 |
| rs62149416^ref25^ | 0.157 | 0.018 | 2.00E-17 |
| rs2082412^ref17^ | 0.365 | 0.033 | 2.00E-28 |
| rs12191877^ref17^ | 0.971 | 0.046 | 1.00E-100 |
| rs20541^ref17^ | 0.239 | 0.031 | 5.00E-15 |
| rs610604^ref17^ | 0.174 | 0.026 | 9.00E-12 |
| rs280519^ref18^ | 0.122 | 0.021 | 4.00E-09 |
| rs3213094^ref18^ | 0.329 | 0.05 | 5.00E-11 |
| rs458017^ref18^ | 0.315 | 0.038 | 2.00E-16 |
| rs27524^ref18^ | 0.122 | 0.018 | 3.00E-11 |
| rs10782001^ref19^ | 0.148 | 0.024 | 9.00E-10 |
| rs12586317^ref19^ | 0.14 | 0.025 | 2.00E-08 |
| rs4795067^ref19^ | 0.174 | 0.026 | 4.00E-11 |
| rs465969^ref21^ | 0.339 | 0.05 | 8.00E-12 |
| rs495337^ref21^ | 0.176 | 0.03 | 3.00E-09 |
| rs12564022^ref22^ | 0.157 | 0.026 | 2.00E-09 |
| rs1990760^ref22^ | 0.166 | 0.029 | 1.00E-08 |
| rs17728338^ref22^ | 0.47 | 0.051 | 6.00E-20 |
| rs2066807^ref22^ | 0.336 | 0.049 | 5.00E-12 |
| rs2853694^ref22^ | 0.207 | 0.033 | 5.00E-10 |
| rs3747517^ref22^ | 0.262 | 0.03 | 1.00E-18 |
| rs4845459^ref22^ | 0.239 | 0.029 | 5.00E-17 |
| rs4921493^ref22^ | 0.207 | 0.029 | 7.00E-13 |
| rs643177^ref22^ | 0.223 | 0.033 | 9.00E-12 |
| rs6590334^ref22^ | 0.131 | 0.022 | 2.00E-09 |
| rs7709212^ref22^ | 0.322 | 0.028 | 2.00E-30 |
| rs842625^ref22^ | 0.148 | 0.023 | 2.00E-10 |
| rs55823223^ref24^ | 0.14 | 0.024 | 1.00E-08 |
| rs11121129^ref25^ | 0.122 | 0.022 | 2.00E-08 |
| rs11652075^ref25^ | 0.104 | 0.019 | 3.00E-08 |
| rs2451258^ref25^ | 0.113 | 0.02 | 3.00E-08 |
| rs367569^ref25^ | 0.122 | 0.022 | 5.00E-08 |
| rs892085^ref25^ | 0.157 | 0.019 | 3.00E-17 |

Supplemental Table 3 continued

| SNP | Beta | SE | *P* |
| --- | --- | --- | --- |
| rs17716942^ref25^ | 0.239 | 0.027 | 3.00E-18 |
| rs2233278^ref25^ | 0.464 | 0.034 | 2.00E-42 |
| rs582757^ref25^ | 0.207 | 0.02 | 2.00E-25 |
| rs7536201^ref25^ | 0.122 | 0.017 | 2.00E-12 |
| rs12188300^ref25^ | 0.457 | 0.03 | 3.00E-53 |
| rs13437088^ref25^ | 0.278 | 0.021 | 3.00E-40 |
| rs27432^ref25^ | 0.182 | 0.02 | 2.00E-20 |
| rs2910686^ref25^ | 0.113 | 0.02 | 2.00E-08 |
| rs33980500^ref25^ | 0.419 | 0.03 | 4.00E-45 |
| rs34536443^ref25^ | 0.631 | 0.055 | 9.00E-31 |
| rs4379175^ref25^ | 0.27 | 0.02 | 9.00E-40 |
| rs4406273^ref25^ | 1.463 | 0 | 5.00E-723 |
| rs6677595^ref25^ | 0.231 | 0.019 | 2.00E-33 |
| rs7552167^ref25^ | 0.191 | 0.028 | 9.00E-12 |

*SNP* single nucleotide polymorphism.

**Supplemental Table 4** The pleiotropic psoriasis-associated SNPs with cardiometabolic traits in European ancestry

| SNP | Trait | *p* value |
| --- | --- | --- |
| rs2395029 | Total cholesterol | 2.46E-05 |
|  | Heart failure | 1.09E-04 |
| rs2082412 | Body mass index | 1.64E-04 |
| rs12191877 | Total cholesterol | 1.47E-04 |
| rs2066808 | Coronary artery disease | 1.85E-04 |
| rs3213094 | Body mass index | 8.64E-05 |
| rs458017 | HDL cholesterol | 3.50E-04 |
| rs10484554 | HDL cholesterol | 1.01E-04 |
| rs27524 | Body mass index | 3.20E-11 |
|  | Obesity | 1.08E-04 |
| rs10782001 | Body mass index | 3.20E-11 |
|  | Diastolic blood pressure | 1.38E-08 |
| rs12586317 | Glycated haemoglobin | 5.15E-05 |
| rs4795067 | Diastolic blood pressure | 4.93E-04 |
| rs2546890 | Body mass index | 3.07E-06 |
| rs1047781 | Type 2 diabetes | 6.20E-06 |
| rs465969 | HDL cholesterol | 5.90E-07 |
| rs1990760 | Coronary artery disease | 4.40E-07 |
| rs2066807 | Coronary artery disease | 1.57E-04 |
|  | HbA1C | 2.90E-08 |
| rs2853694 | Systolic blood pressure | 2.20E-04 |
| rs34394770 | HbA1C | 1.20E-05 |
| rs3747517 | HDL cholesterol | 7.26E-06 |
| rs6590334 | Coronary artery disease | 3.10E-04 |
|  | Myocardial infarction | 3.00E-04 |
| rs7709212 | Body mass index | 2.77E-06 |
| rs8128234 | Diastolic blood pressure | 3.97E-08 |
|  | Systolic blood pressure | 2.03E-05 |
| rs2675662 | Atrial fibrillation | 1.20E-06 |
|  | Diastolic blood pressure | 2.11E-08 |
|  | Type 2 diabetes | 4.91E-07 |
| rs11059675 | Body mass index | 1.10E-08 |
|  | Diastolic blood pressure | 4.03E-05 |
| rs11065979 | Body mass index | 1.40E-06 |
|  | Cardiovascular diseases | 1.32E-08 |
| rs28624578 | HDL cholesterol | 3.51E-07 |
| rs2944542 | Body mass index | 4.60E-04 |
| rs34517439 | Body mass index | 3.60E-37 |
|  | Diastolic blood pressure | 2.02E-19 |

**Supplemental Table 4** continued

| SNP | Trait | *p* value |
| --- | --- | --- |
| rs492602 | Diastolic blood pressure | 9.10E-06 |
| rs55823223 | Body mass index | 3.10E-08 |
|  | Diastolic blood pressure | 1.13E-09 |
|  | Type 2 diabetes | 1.63E-04 |
| rs559406 | Body mass index | 2.60E-04 |
| rs61871342 | Diabetes diagnosed by doctor | 2.50E-08 |
| rs11121129 | Cholesterol | 8.00E-05 |
| rs2451258 | HDL cholesterol | 1.78E-05 |
| rs367569 | Cholesterol | 2.28E-05 |
| rs545979 | Coronary artery disease | 8.60E-05 |
|  | Diastolic blood pressure | 9.27E-10 |
| rs892085 | Body mass index | 2.80E-05 |
|  | Ischemic stroke | 8.43E-05 |
|  | Coronary artery disease | 1.39E-04 |
| rs963986 | HDL cholesterol | 7.33E-08 |
| rs11795343 | HDL cholesterol | 4.29E-05 |
|  | Type 2 diabetes | 1.31E-04 |
| rs12720356 | Body mass index | 3.20E-04 |
| rs17716942 | Coronary artery disease | 4.79E-05 |
| rs4561177 | HDL cholesterol | 1.90E-06 |
| rs8016947 | Body mass index | 8.53E-05 |
| rs12445568 | Body mass index | 5.00E-12 |
| rs13437088 | Cholesterol | 3.08E-19 |
| rs2066819 | Coronary artery disease | 1.42E-04 |
| rs2111485 | Coronary artery disease | 2.41E-07 |
| rs27432 | Body mass index | 1.70E-05 |
| rs2910686 | Coronary artery disease | 5.45E-05 |
|  | Diastolic blood pressure | 1.51E-12 |
| rs34536443 | Cholesterol | 2.74E-04 |
| rs4379175 | Body mass index | 3.23E-05 |
| rs4406273 | Cholesterol | 7.55E-10 |
| rs4821124 | Cholesterol | 1.08E-14 |
| rs7552167 | Triglycerides | 7.12E-06 |

*SNP* single nucleotide polymorphism, *HDL* high-density lipoprotein, *HbA1C* glycosylated hemoglobin.

**Supplemental Table 5** Heterogeneity and pleiotropy analysis of the psoriasis on CAD, MI and HF risk.

| Trait | MR Method | MR Result in Europeans | | | |  | MR Result in East Asian | | | |
| --- | --- | --- | --- | --- | --- | --- | --- | --- | --- | --- |
|  |  | Cochran Q statistic | Heterogeneity  *p* value | Intercept | Intercept *p* value |  | Cochran Q statistic | Heterogeneity  *p* value | Intercept | Intercept *p* value |
| CAD | Inverse variance weighted | 4.66 | 0.990 | 0.0006 | 0.896 |  | 10.02 | 0.528 | 0.054 | 0.049 |
|  | MR Egger | 4.64 | 0.982 |  |  |  | 4.65 | 0.913 |  |  |
| MI | Inverse variance weighted | 11.19 | 0.798 | 0.003 | 0.699 |  | 8.22 | 0.768 | 0.009 | 0.255 |
|  | MR Egger | 11.03 | 0.750 |  |  |  | 6.77 | 0.818 |  |  |
| HF | Inverse variance weighted | 22.16 | 0.138 | 0.003 | 0.731 |  | 10.36 | 0.584 | 0.003 | 0.718 |
|  | MR Egger | 21.98 | 0.108 |  |  |  | 10.22 | 0.510 |  |  |

*MR* Mendelian randomization*,* *CAD* coronary artery disease, *MI* myocardial infarction, *HF* heart failure.

**Supplemental Table 6** SNPs (*p* < 5 X 10^-8^) associated with psoriasis of East Asian ancestry were previous reported

| SNP | Beta | SE | *P* |
| --- | --- | --- | --- |
| rs1050414^ref30^ | 1.161 | 0.155 | 6.00E-14 |
| rs11544355^ref29^ | -1.772 | 0.272 | 7.00E-11 |
| rs12884468^ref28^ | -0.128 | 0.022 | 1.00E-08 |
| rs143700362^ref29^ | -3.507 | 0.391 | 3.00E-19 |
| rs144706178^ref29^ | -2.207 | 0.278 | 2.00E-15 |
| rs149442660^ref29^ | -3.219 | 0.468 | 6.00E-12 |
| rs149798287^ref29^ | -0.994 | 0.173 | 1.00E-08 |
| rs2233278^ref27^ | 0.673 | 0.108 | 4.00E-10 |
| rs2276405^ref28^ | -0.186 | 0.031 | 3.00E-09 |
| rs249038^ref28^ | -0.174 | 0.031 | 2.00E-08 |
| rs2778031^ref28^ | -0.186 | 0.015 | 1.00E-36 |
| rs2781377^ref28^ | -0.163 | 0.025 | 4.00E-11 |
| rs41268474^ref28^ | 0.157 | 0.024 | 6.00E-11 |
| rs4141001^ref28^ | -0.151 | 0.022 | 2.00E-11 |
| rs5063^ref28^ | -0.163 | 0.028 | 3.51E-09 |
| rs6444895^ref28^ | 0.104 | 0.015 | 1.00E-12 |
| rs72933970^ref28^ | 0.148 | 0.026 | 1.00E-08 |
| rs9808753^ref28^ | -0.083 | 0.015 | 3.00E-08 |
| rs671^ref28^ | 0.145 | 0.018 | 1.00E-15 |
| rs1042636^ref28^ | 0.094 | 0.015 | 2.00E-10 |
| rs1265181^ref26^ | 3.119 | 0 | 2.00E-208 |
| rs76337351^ref28^ | 0.186 | 0.033 | 2.00E-08 |
| rs12602912^ref28^ | 0.081 | 0.014 | 2.00E-08 |
| rs3213094^ref26^ | 0.247 | 0.023 | 3.00E-26 |
| rs610037^ref28^ | 0.104 | 0.016 | 4.00E-11 |
| rs10036748^ref28^ | 0.095 | 0.016 | 4.00E-09 |
| rs1473247^ref28^ | 0.131 | 0.02 | 6.00E-11 |
| rs9394026^ref27^ | 0.621 | 0.08 | 7.00E-15 |
| rs4085613^ref26^ | 0.278 | 0.024 | 7.00E-30 |
| rs27044^ref28^ | 0.134 | 0.014 | 8.00E-21 |
| rs3174808^ref28^ | 0.095 | 0.017 | 1.00E-08 |
| rs35960711^ref28^ | 0.14 | 0.009 | 4.00E-53 |
| rs10888501^ref28^ | 0.148 | 0.021 | 6.00E-13 |

*SNP* single nucleotide polymorphism.

**Supplemental Table 7** The pleiotropic psoriasis-associated SNPs with cardiometabolic traits in East Asian ancestry

| SNP | Trait | *p* value |
| --- | --- | --- |
| rs671 | Ischemic stroke | 2.40E-18 |
|  | Arrhythmia stratified | 2.80E-13 |
|  | Type 2 diabetes | 8.30E-05 |
| rs1042636 | Type 2 diabetes | 3.90E-07 |
| rs12602912 | Body mass index | 3.50E-08 |
| rs3213094 | Body mass index | 8.60E-05 |
| rs610037 | Coronary artery disease | 5.70E-05 |
| rs1473247 | Body mass index | 5.40E-04 |
| rs9394026 | Coronary artery disease | 2.60E-05 |
| rs27044 | Ischemic stroke | 1.40E-03 |

*SNP* single nucleotide polymorphism.

**Supplemental Table 8** MR Steiger directionality test

| Ancestry | **Exposure** | **Outcome** | **snp_R^2^.exposure** | **snp_R^2^.outcome** | **correct_causal_direction** |
| --- | --- | --- | --- | --- | --- |
| European population | psoriasis | CAD | 0.023896 | 2.26E-05 | TRUE |
|  | psoriasis | MI | 0.026605 | 0.0001748 | TRUE |
|  | psoriasis | HF | 0.026605 | 6.50E-05 | TRUE |
| East Asian population | psoriasis | CAD | 0.027294 | 0.0002851 | TRUE |
|  | psoriasis | MI | 0.02762 | 6.78E-05 | TRUE |
|  | psoriasis | HF | 0.02762 | 6.80E-05 | TRUE |

R^2^ values are approximate; correct_causal_direction: the inferred causal direction between exposures and outcome.

*MR* Mendelian randomization*, CAD* coronary artery disease, *MI* myocardial infarction, *HF* heart failure.

**Supplementary Figure 1** Sensitivity analysis of the meta-analyses about psoriasis and CAD risk (a), MI risk (b) and HF risk (c). CAD, coronary artery disease; MI, myocardial infarction; HF, heart failure.

(a)


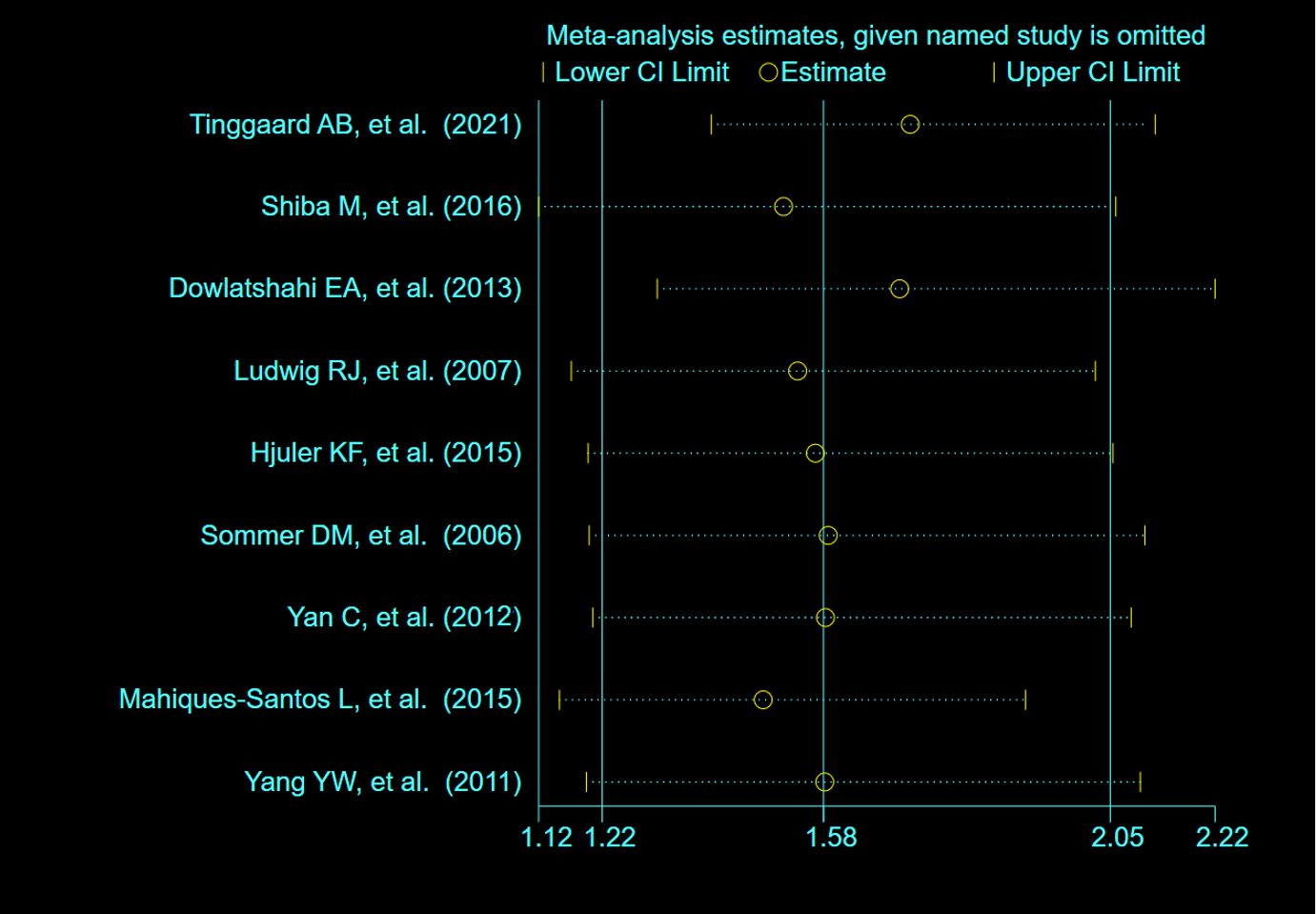


(b)


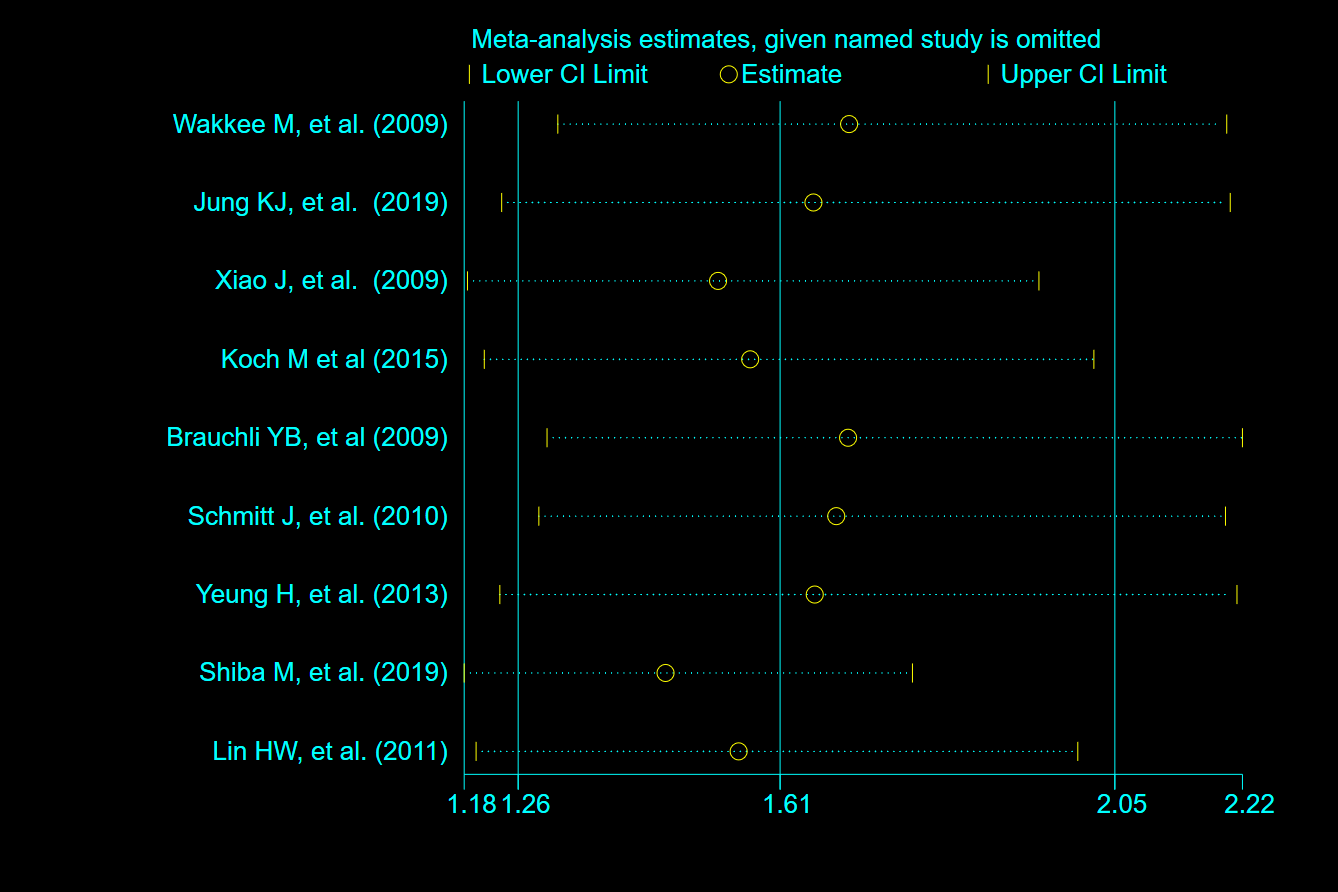


(c)


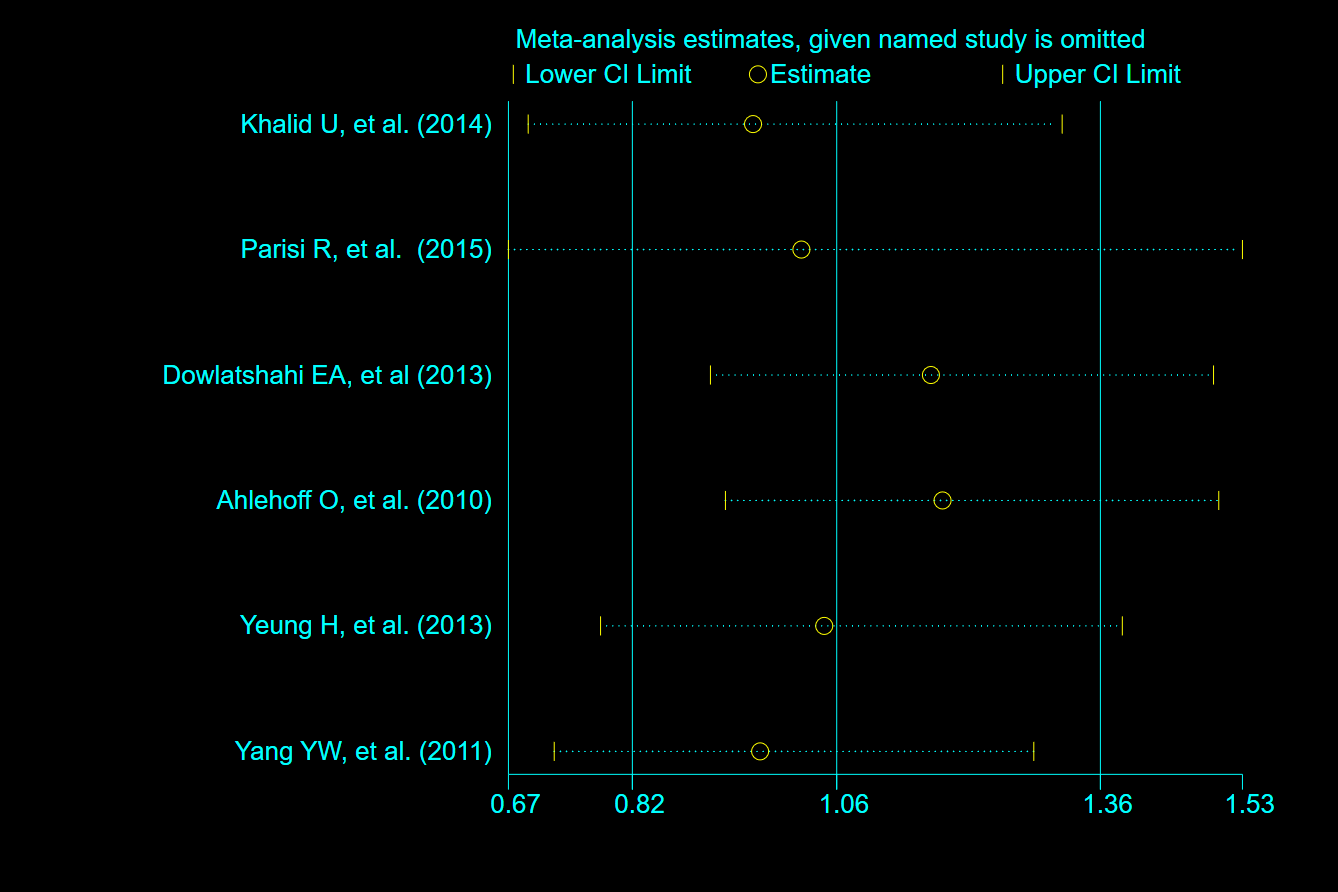


**Supplementary Figure 2**

A plot relating the effect sizes of the SNP-psoriasis association (x-axis, log OR) and the SNP-CVD associations (y-axis, log OR) with standard error bars. The plot relating the effect sizes of the SNP-psoriasis association and the SNP-CAD (a), SNP-MI (b) and SNP-HF (c) in European population and the plot relating the effect sizes of the SNP-psoriasis association and the SNP-CAD (d), SNP-MI (e) and SNP-HF (f) in East Asian population. The slopes of the lines correspond to causal estimates using each of the five different methods.

(a)


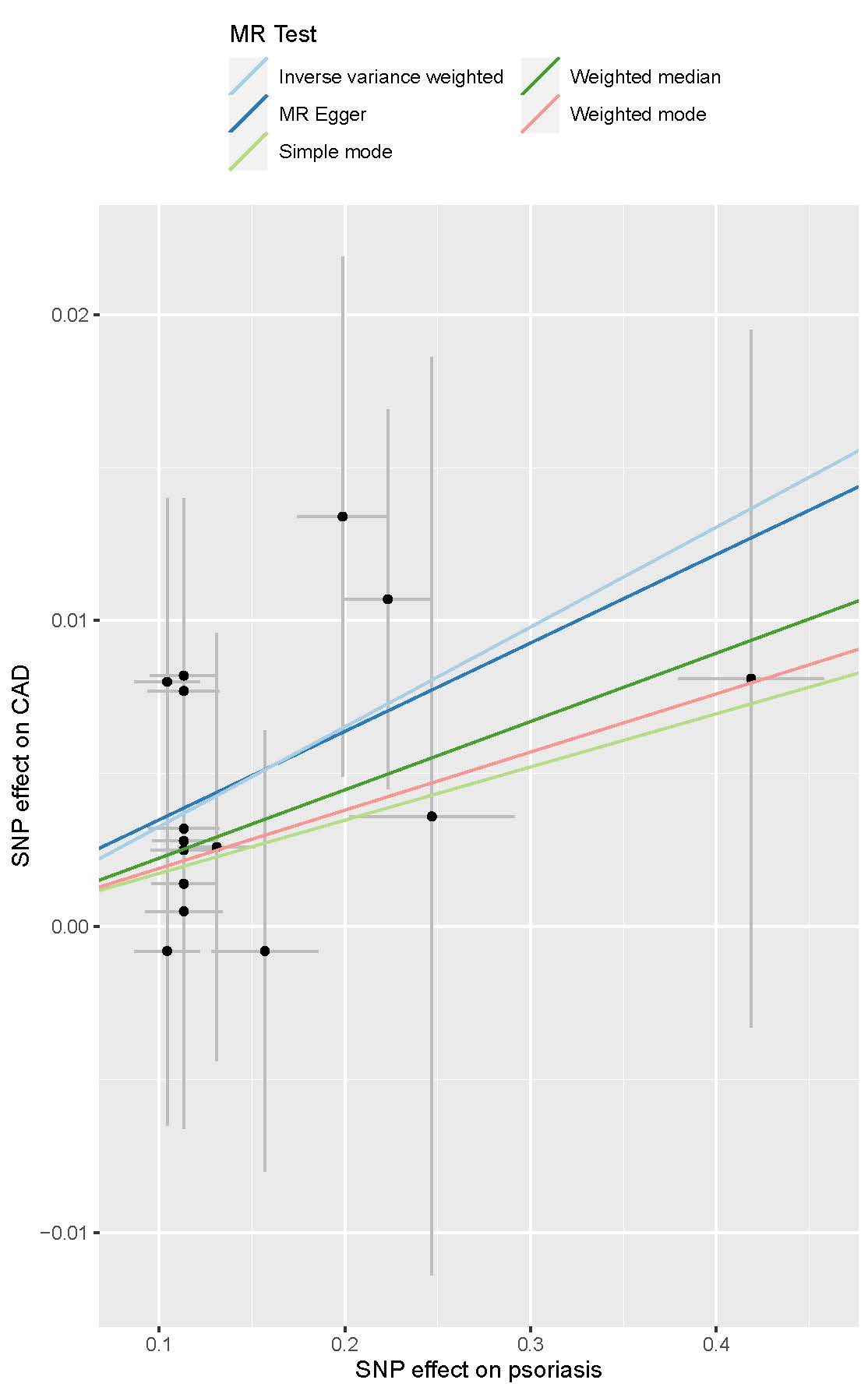


(b)


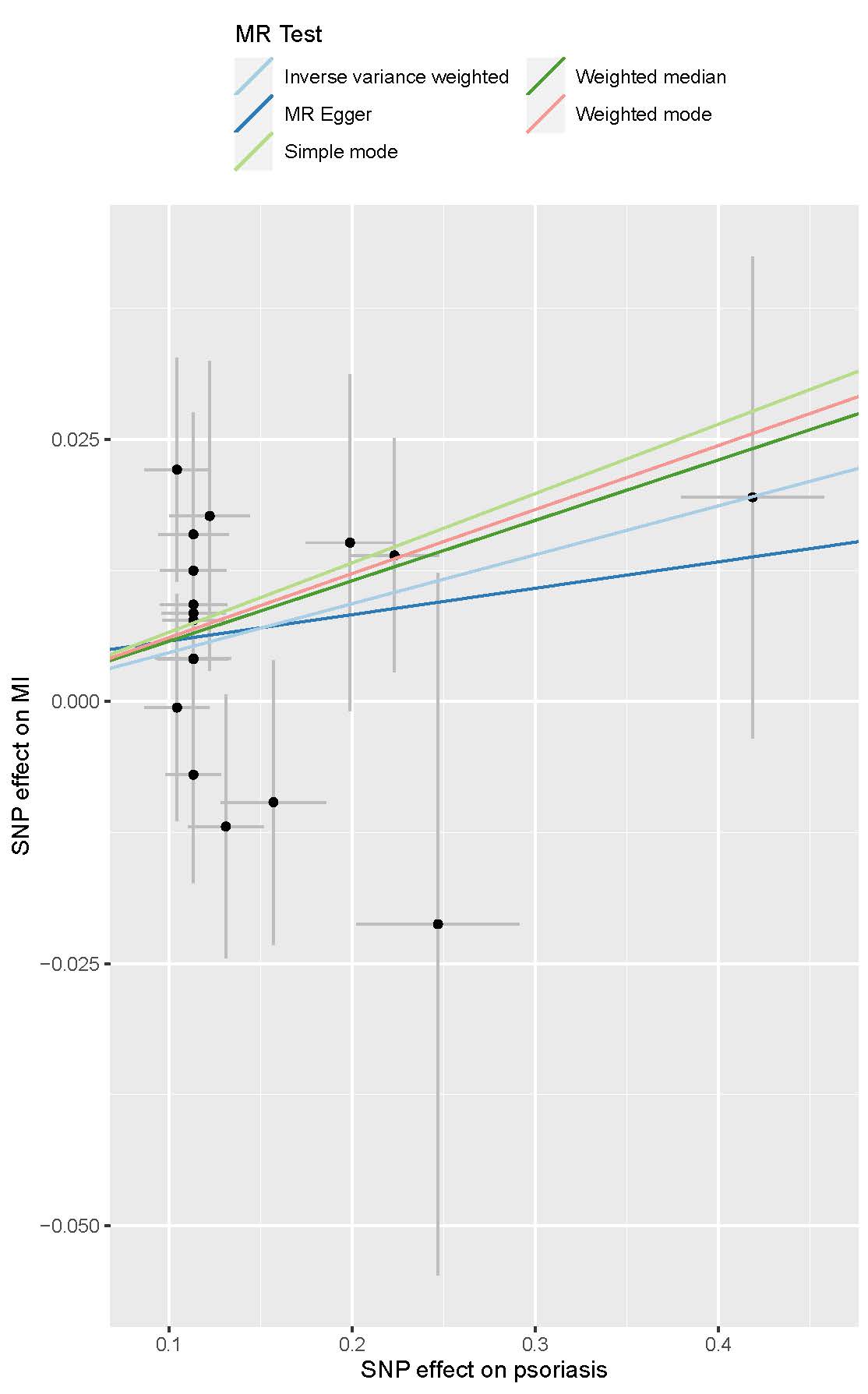


(c)


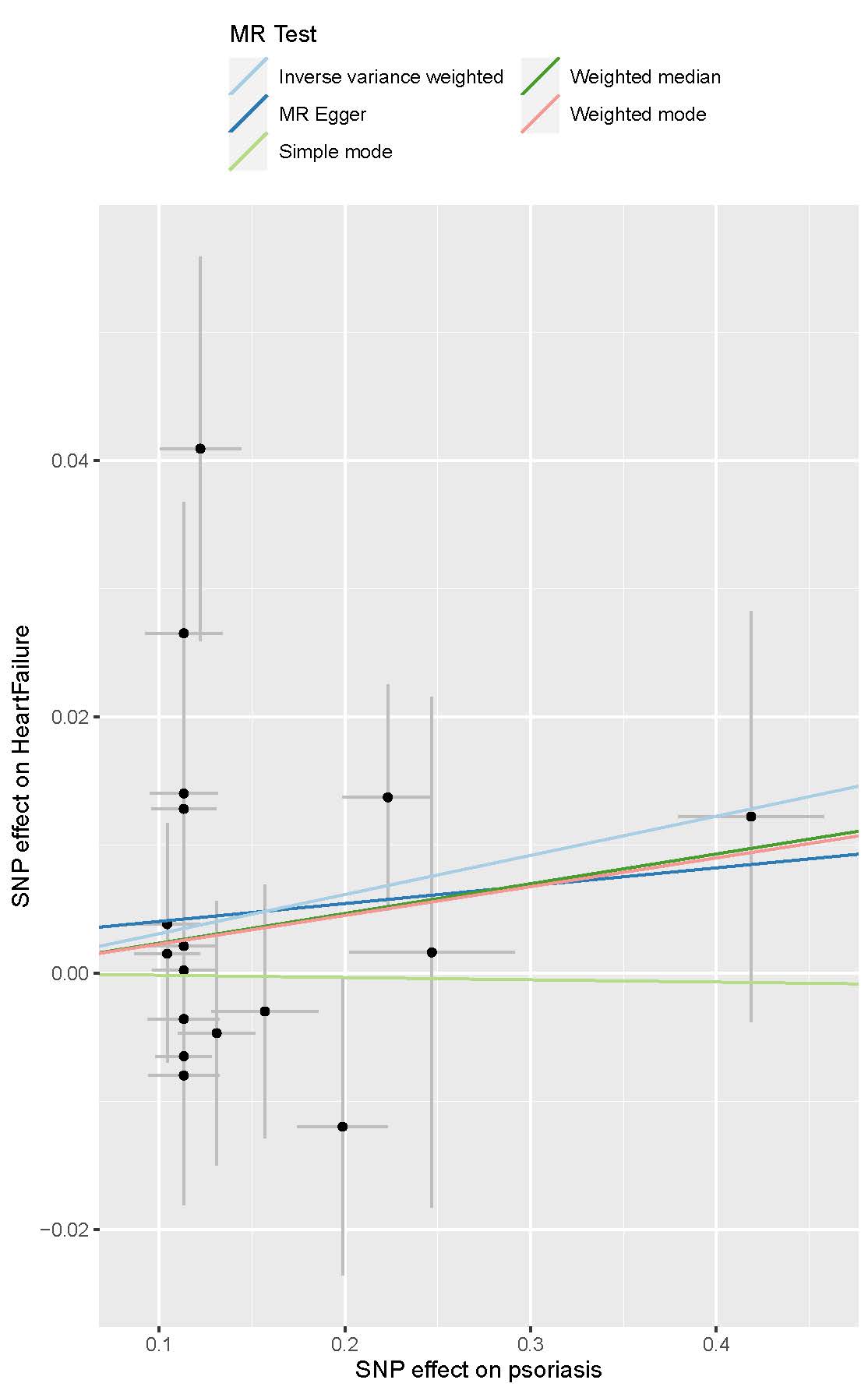


(d)


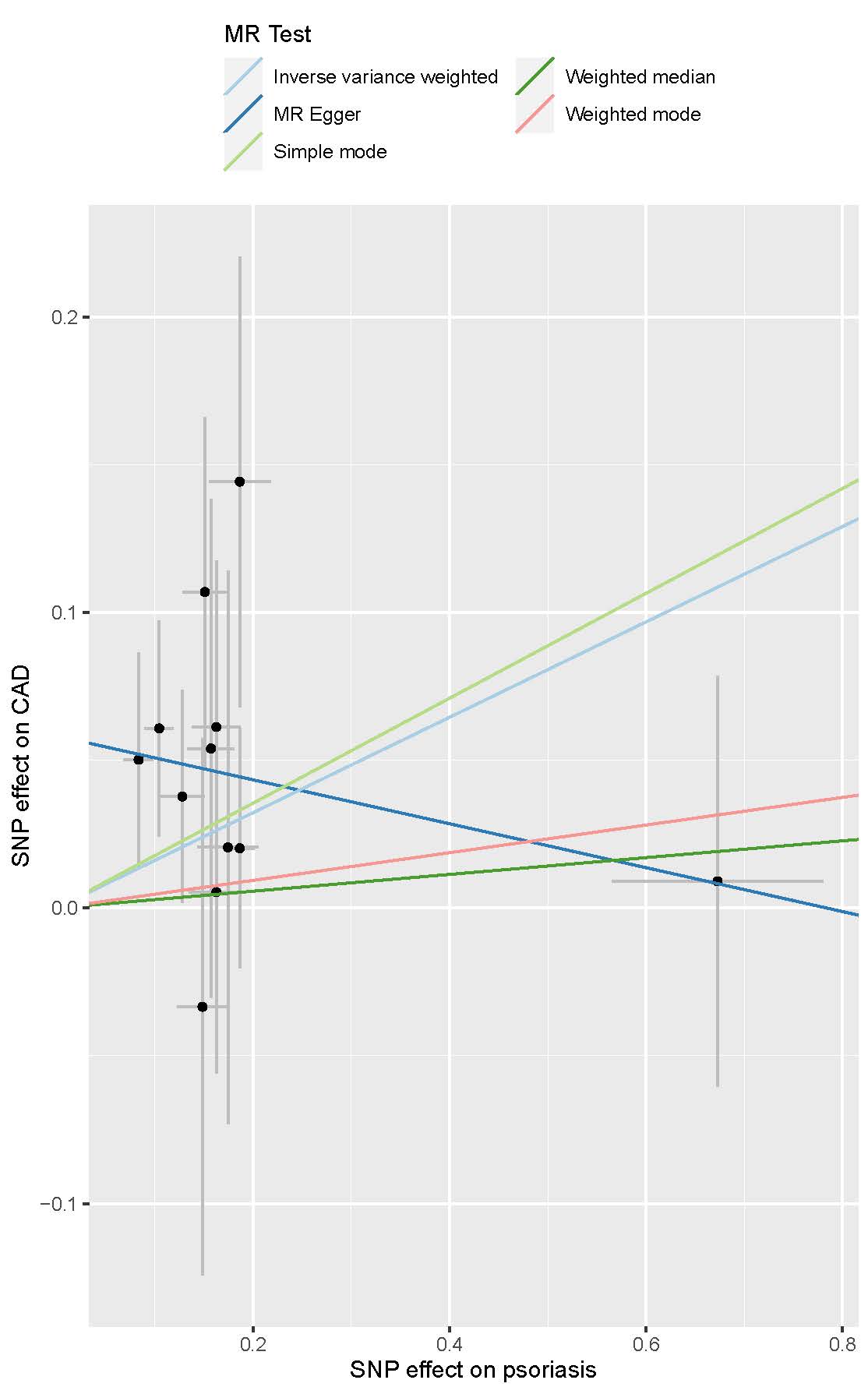


(e)


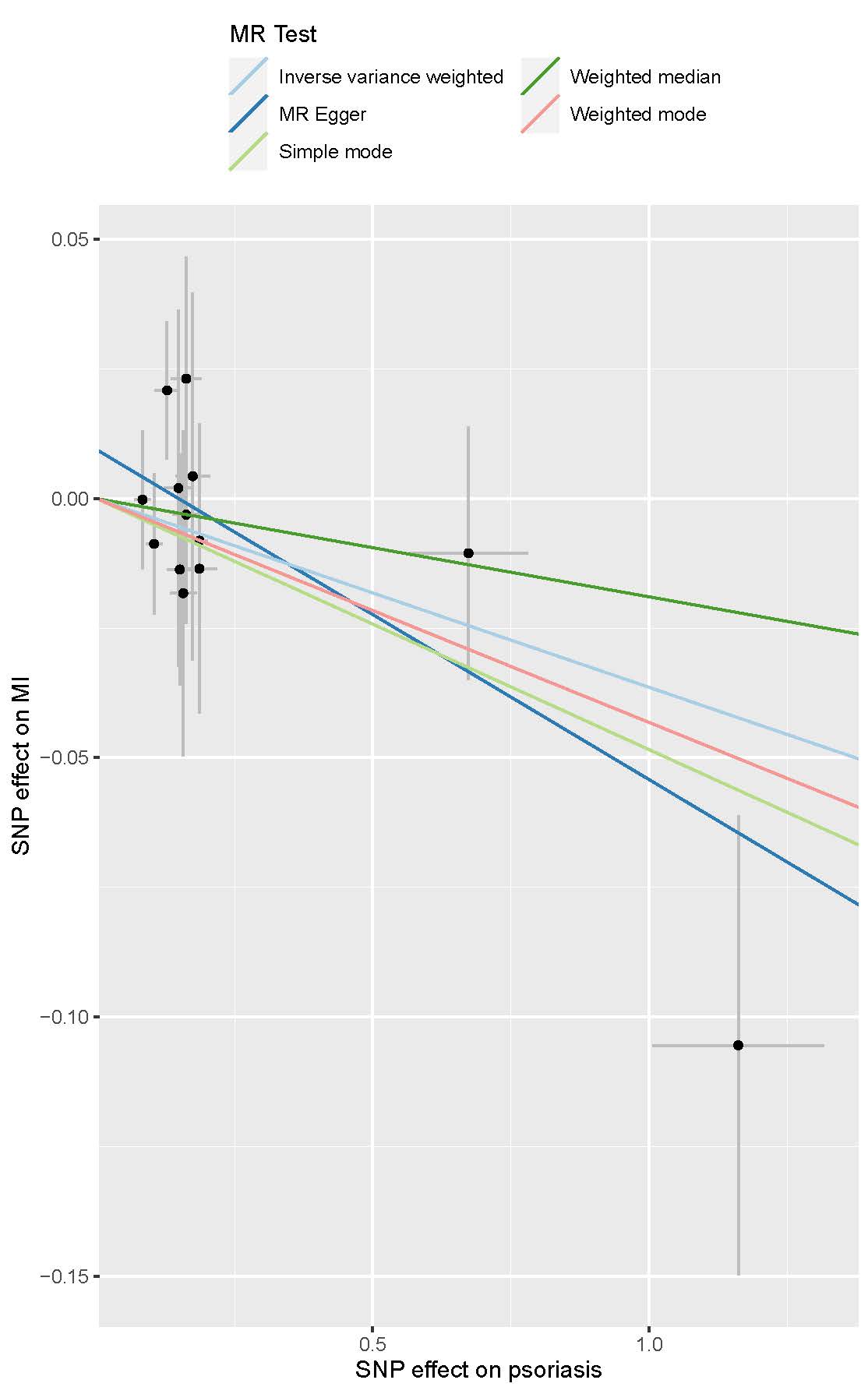


(f)


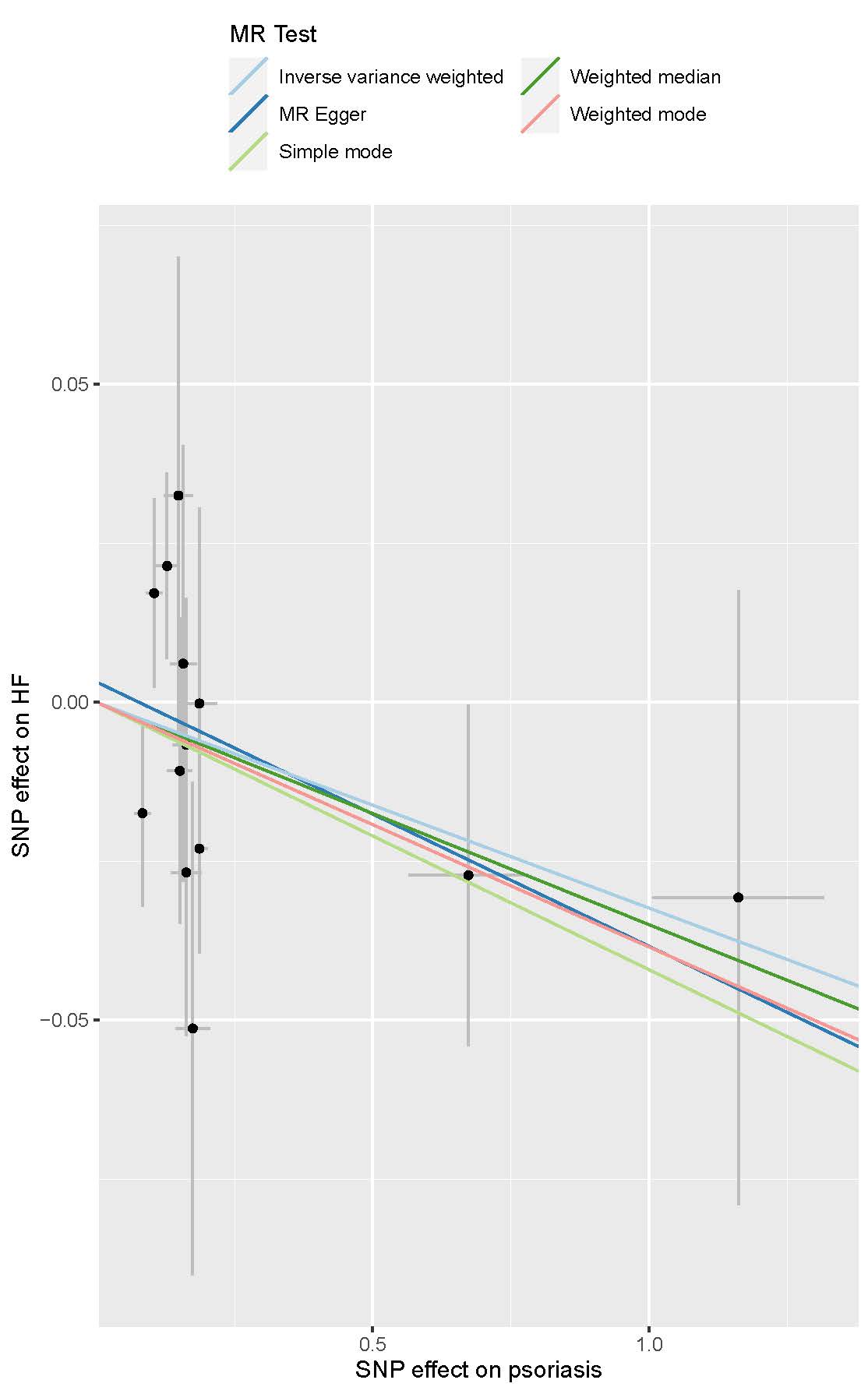


**Supplementary Figure 3** Leave-one-out MR analysis for SNPs used as instruments MR analysis (black). The impact of psoriasis on CAD (a), MI (b) and HF (c) in European population and the impact of psoriasis on CAD (d), MI (e) and HF (f) in East Asian population. MR, Mendelian randomization; SNP, single nucleotide polymorphism, CAD, coronary artery disease; MI, myocardial infarction; HF, heart failure.

(a)


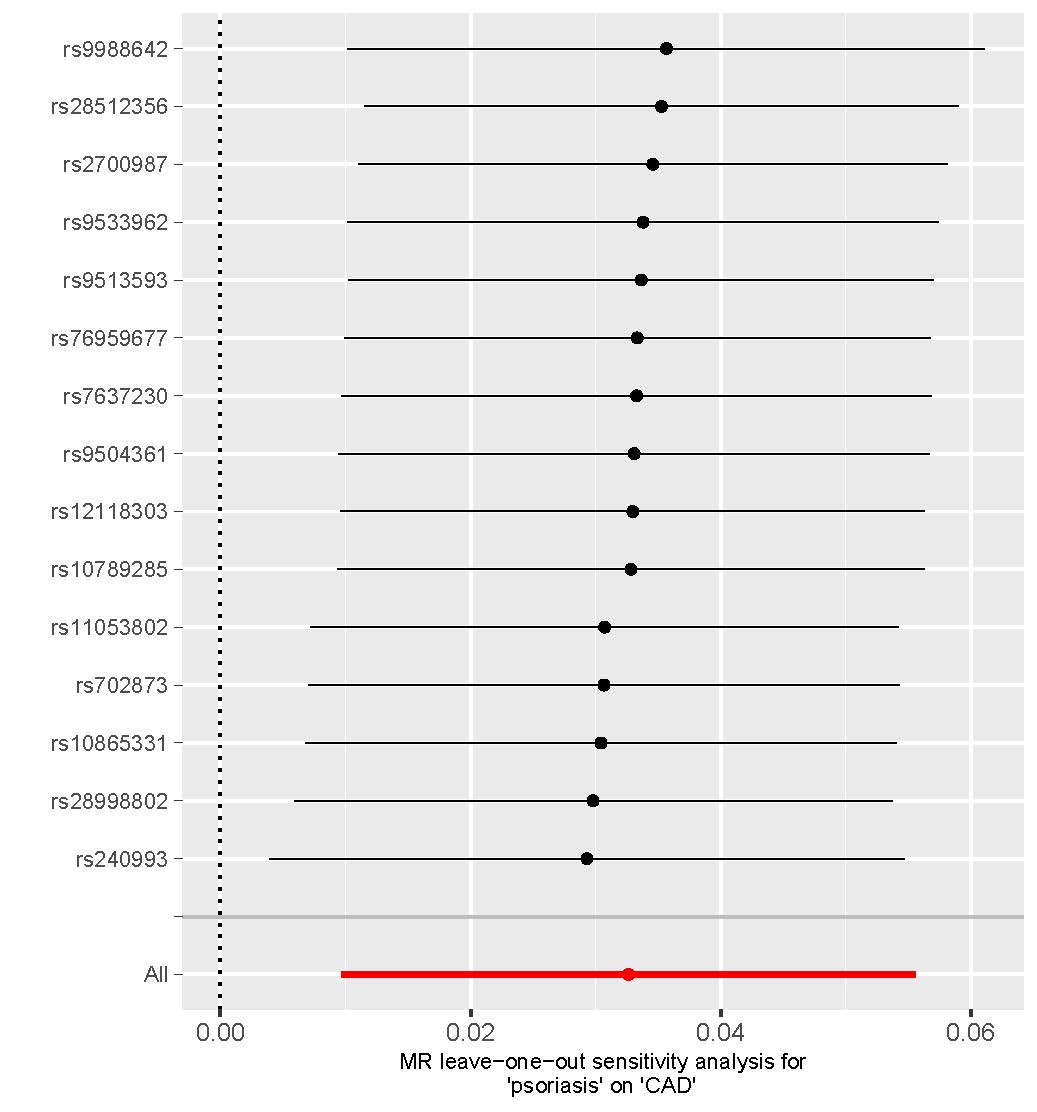


(b)


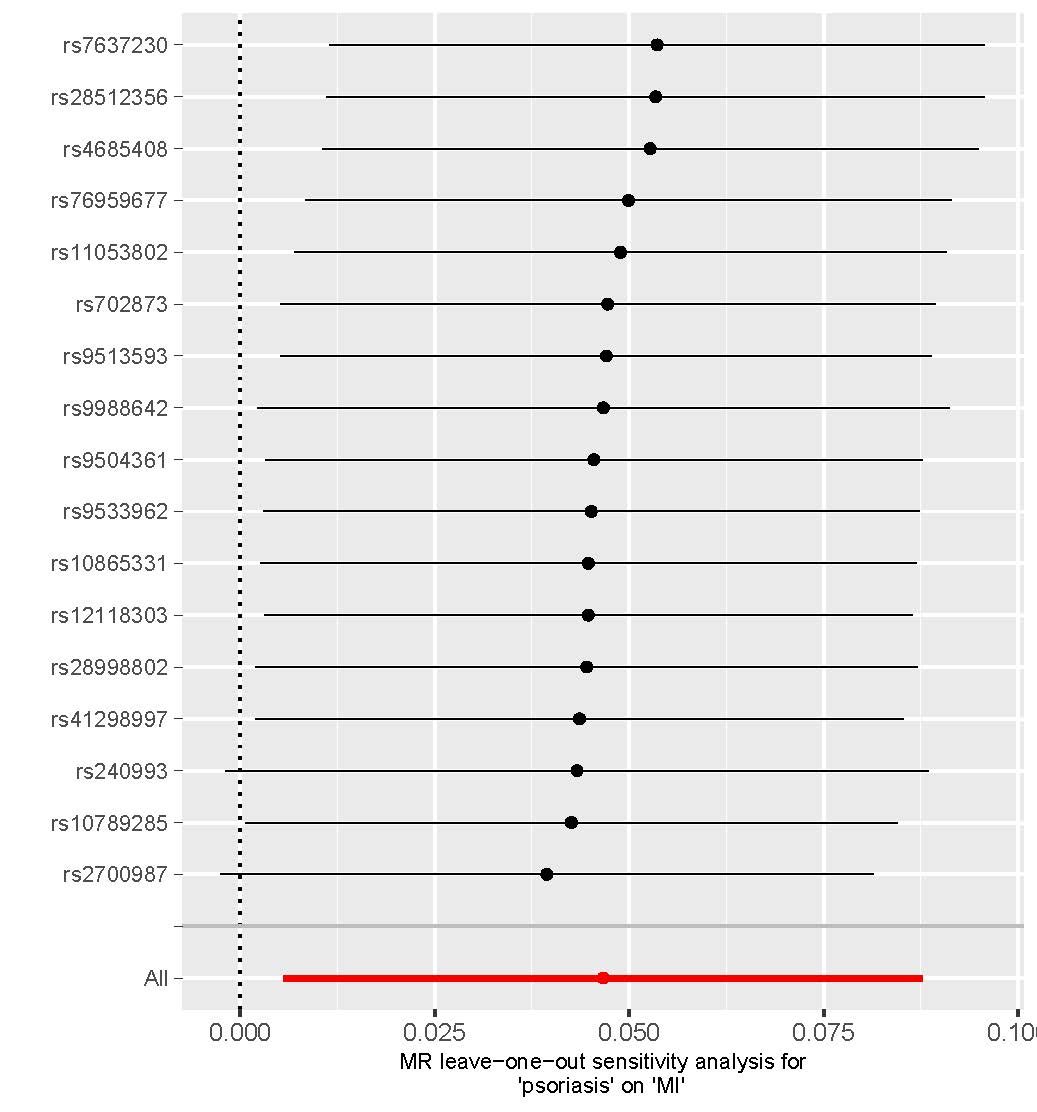


(c)


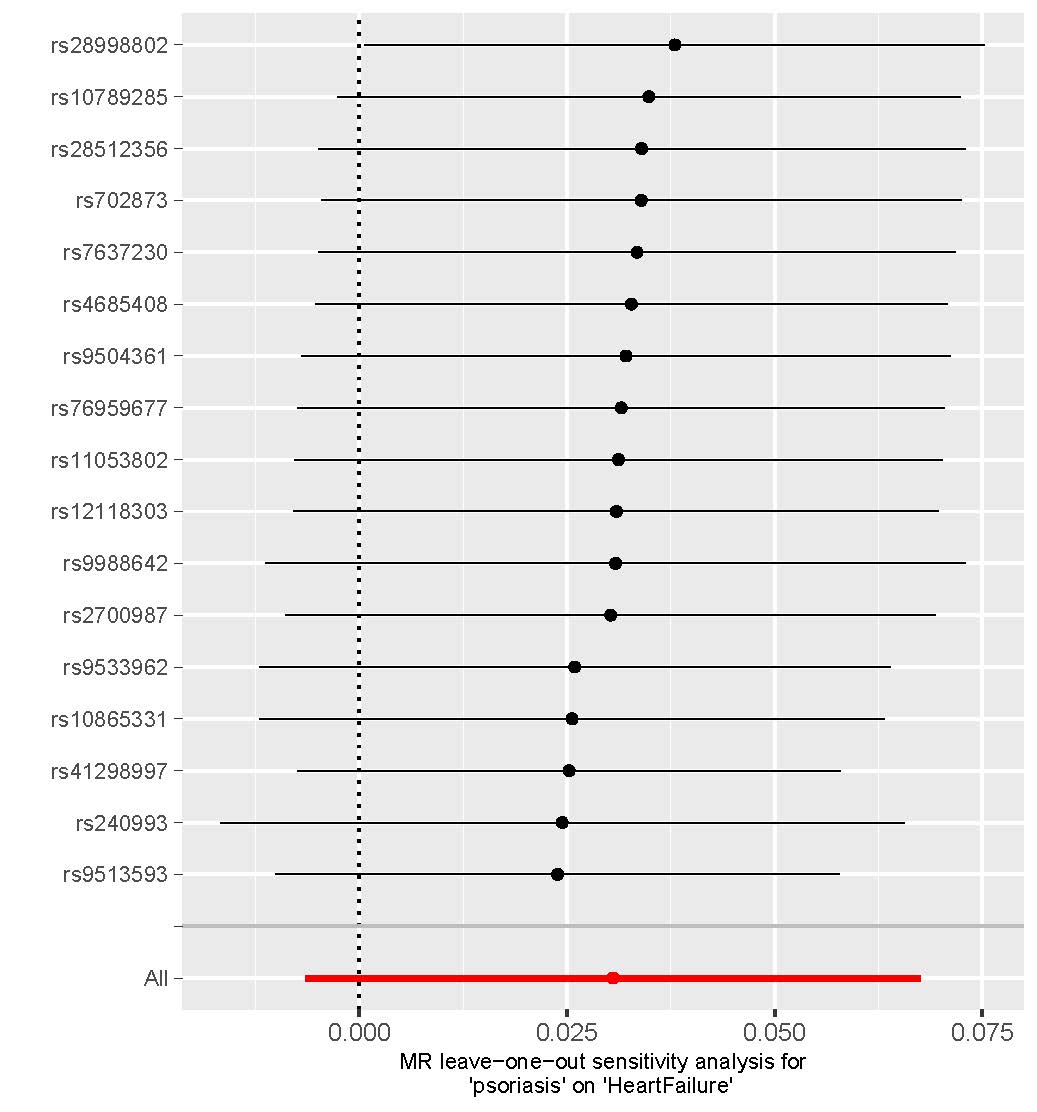


(d)


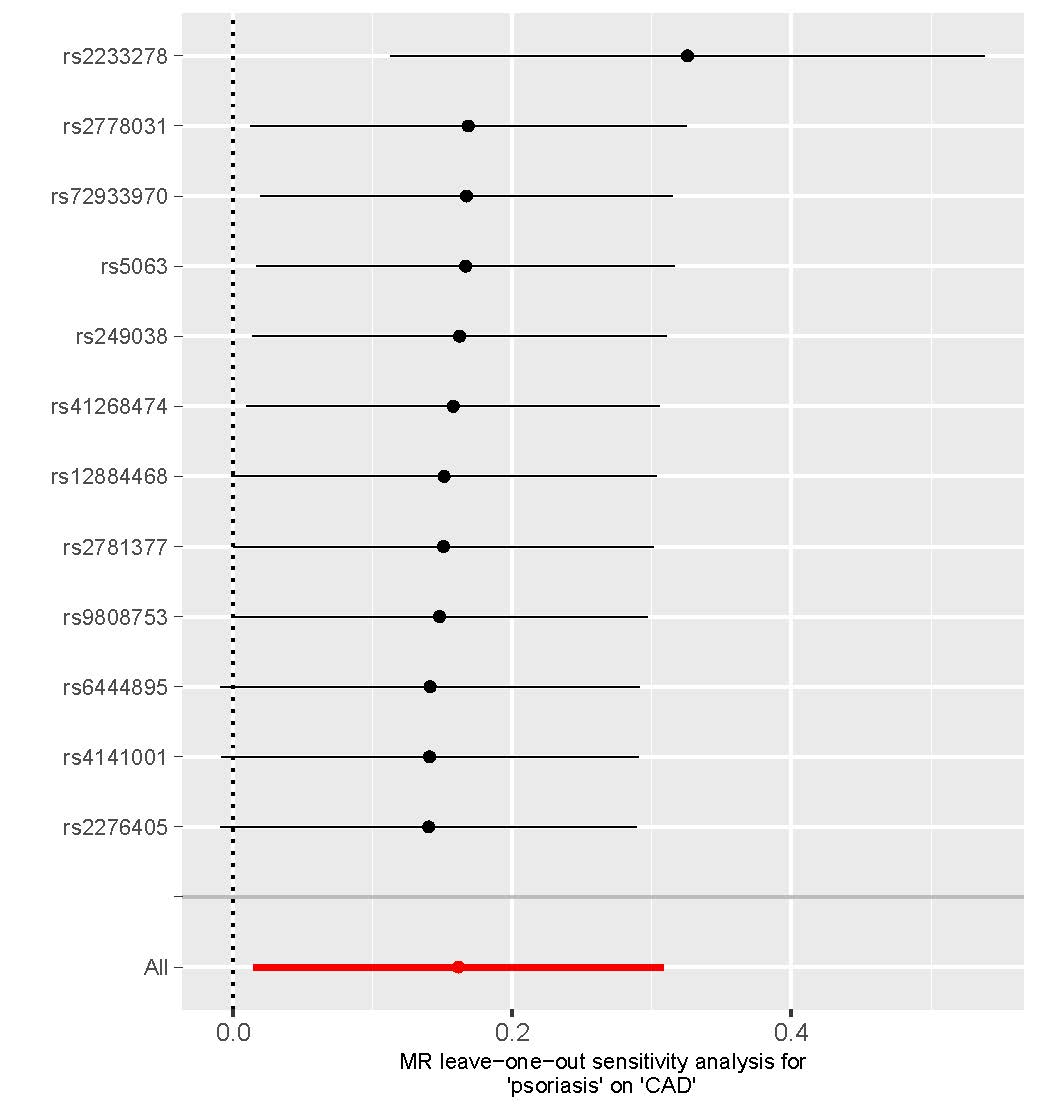


(e)


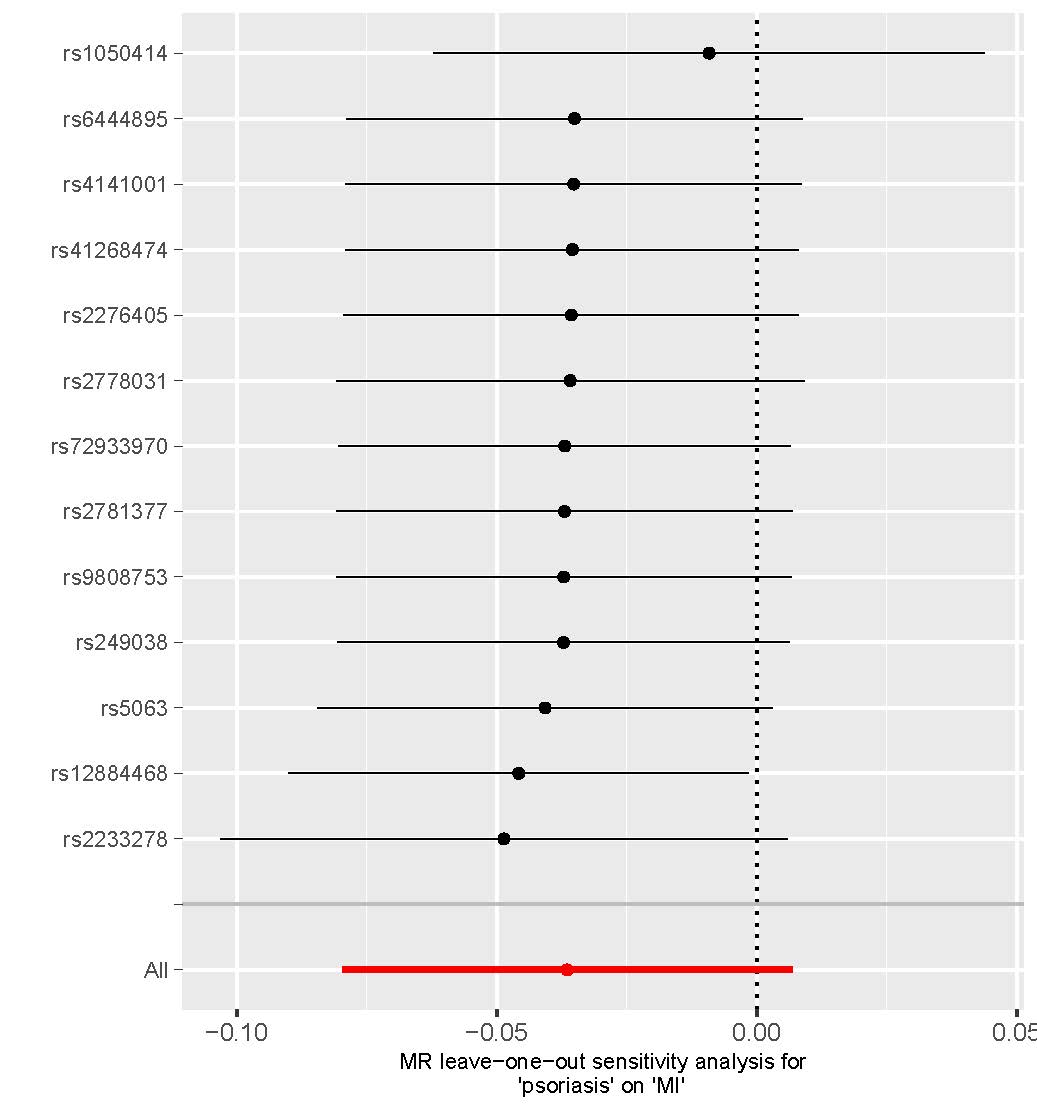


(f)


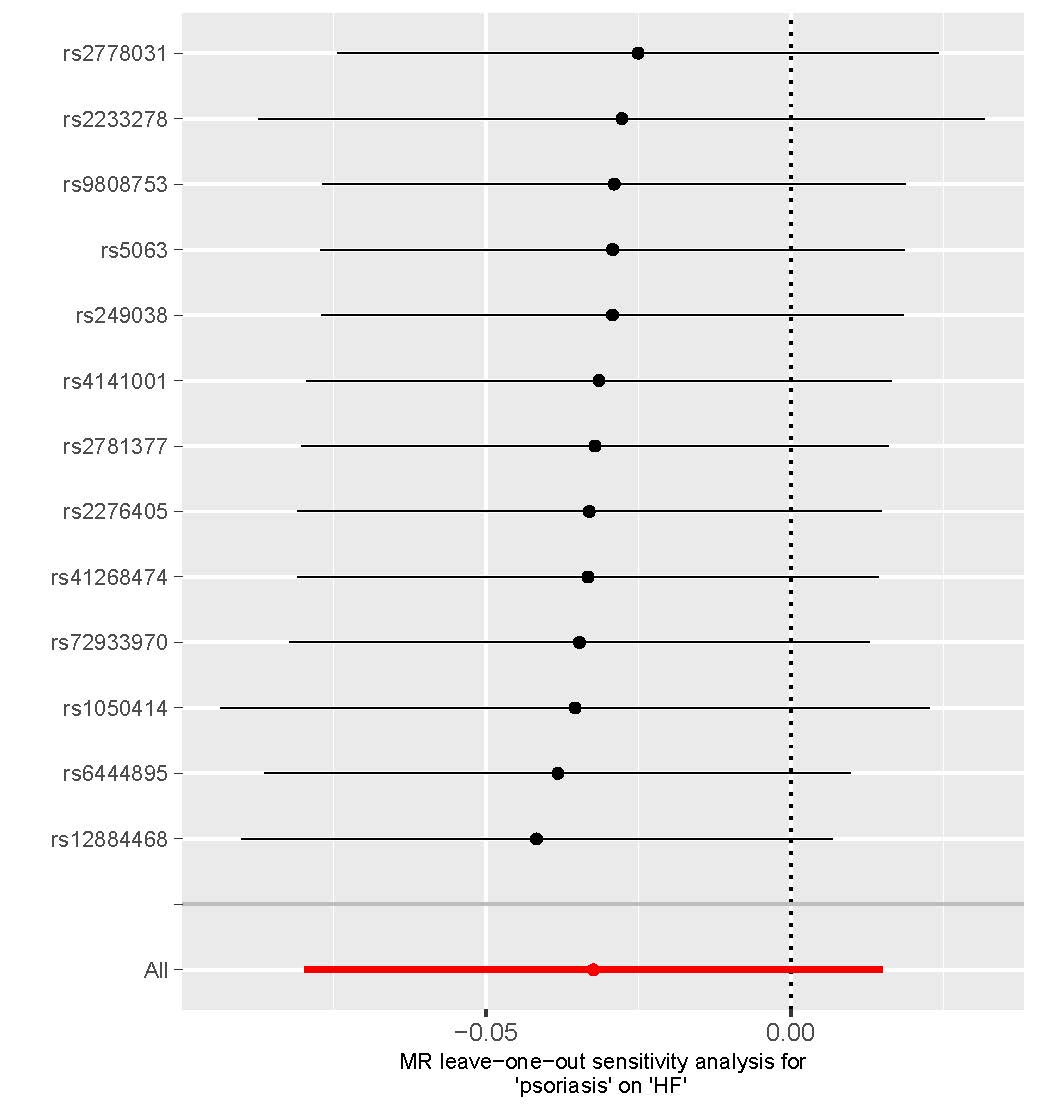

Supplement: Supplementary file 1 — Additional file 1: Table S1. Search strategy of psoriasis and coronary artery disease. Table S2. Characteristics and methodological quality of the qualified studies of the 3 meta-analyses. Table S3. SNPs (p < 5 × 10^−8^) associated with psoriasis of European ancestry were previous reported. Table S4. The pleiotropic psoriasis-associated SNPs with cardiometabolic traits in European ancestry. Table S5. Heterogeneity and pleiotropy analysis of the psoriasis on CAD, MI and HF risk. Table S6. SNPs (p < 5 × 10^−8^) associated with psoriasis of East Asian ancestry were previous reported. Table S7. The pleiotropic psoriasis-associated SNPs with cardiometabolic traits in East Asian ancestry. Table S8. MR Steiger directionality test. Figure S1. Sensitivity analysis of the meta-analyses about psoriasis and CAD risk (a), MI risk (b) and HF risk (c). Figure S2. A plot relating the effect sizes of the SNP-psoriasis association (x-axis, log OR) and the SNP-CVD associations (y-axis, log OR) with standard error bars. Figure S3. Leave-one-out MR analysis for SNPs used as instruments MR analysis. [file 12916_2022_2617_MOESM1_ESM.docx]
